# Multi-layered proteomics identifies insulin-induced upregulation of the EphA2 receptor via the ERK pathway which is dependent on low IGF1R level

**DOI:** 10.1038/s41598-024-77817-5

**Published:** 2024-11-21

**Authors:** Sarah Hyllekvist Jørgensen, Kristina Bennet Emdal, Anna-Kathrine Pedersen, Lene Nygaard Axelsen, Helene Faustrup Kildegaard, Damien Demozay, Thomas Åskov Pedersen, Mads Grønborg, Rita Slaaby, Peter Kresten Nielsen, Jesper Velgaard Olsen

**Affiliations:** 1 Proteomics Program, Novo Nordisk Foundation Center for Protein Research, Faculty of Health and Medical Sciences, University of Copenhagen, 2200 Copenhagen, Denmark; 2 Global Research Technologies, Novo Nordisk A/S, 2760 Maaloev, Denmark; 3 Global Translation, Novo Nordisk A/S, 2760 Maaloev, Denmark; 4 Global Nucleic Acid Therapies, Novo Nordisk A/S, 2760 Maaloev, Denmark; 5 Global Drug Discovery, Novo Nordisk A/S, 2760 Maaloev, Denmark

**Keywords:** Proteomic analysis, Mass spectrometry, Phosphorylation, Protein-protein interaction networks

## Abstract

Insulin resistance impairs the cellular insulin response, and often precedes metabolic disorders, like type 2 diabetes, impacting an increasing number of people globally. Understanding the molecular mechanisms in hepatic insulin resistance is essential for early preventive treatments. To elucidate changes in insulin signal transduction associated with hepatocellular resistance, we employed a multi-layered mass spectrometry-based proteomics approach focused on insulin receptor (IR) signaling at the interactome, phosphoproteome, and proteome levels in a long-term hyperinsulinemia-induced insulin-resistant HepG2 cell line with a knockout of the insulin-like growth factor 1 receptor (IGF1R KO). The analysis revealed insulin-stimulated recruitment of the PI3K complex in both insulin-sensitive and -resistant cells. Phosphoproteomics showed attenuated signaling via the metabolic PI3K-AKT pathway but sustained extracellular signal-regulated kinase (ERK) activity in insulin-resistant cells. At the proteome level, the ephrin type-A receptor 2 (EphA2) showed an insulin-induced increase in expression, which occurred through the ERK signaling pathway and was concordantly independent of insulin resistance. Induction of EphA2 by insulin was confirmed in additional cell lines and observed uniquely in cells with high IR-to-IGF1R ratio. The multi-layered proteomics dataset provided insights into insulin signaling, serving as a resource to generate and test hypotheses, leading to an improved understanding of insulin resistance.

**Supplementary Information:**

The online version contains supplementary material available at 10.1038/s41598-024-77817-5.

## Introduction

Insulin resistance, a hallmark of type 2 diabetes, is characterized by impaired cellular responses to insulin^1,2^. It affects nearly 40% of young American adults^3^ and is influenced by various factors, including obesity, physical inactivity, chronic inflammation, prolonged exposure to elevated levels of nutrients, and pathophysiological insulin levels^4,5^. The cellular response and mechanisms of insulin resistance vary by tissue. However, fat, muscle, and liver serve as the primary insulin-responsive tissues^1^.

The liver plays a key role in glucose homeostasis and overall metabolism, and insulin signaling in hepatocytes primarily coordinates glucose uptake, storage, and production^6^. Insulin binds to the insulin receptor (IR), a plasma membrane-spanning receptor tyrosine kinase (RTK), leading to its autophosphorylation and activation of downstream signaling. Insulin signaling branches into two major pathways - a metabolic pathway via the phosphatidylinositol 3-kinase (PI3K)-AKT signaling axis, which plays the primary role in hepatic insulin response, and a mitogenic pathway controlling cell growth, proliferation, and gene expression^7–10^. The growth factor capabilities of insulin depend on signaling activation via the canonical extracellular signal-regulated kinase (ERK) pathway^8^. Hepatic insulin resistance primarily impairs PI3K-AKT signaling^2^. The relationship between IR levels and insulin resistance is unclear, with some studies reporting reduced IR levels in insulin-resistant states^11–14^ and others observing no significant differences between insulin-sensitivity and insulin-resistance^15,16^.

Quantitative mass spectrometry (MS)-based proteomics is a powerful technology for global analysis of cell signaling networks, and it is widely used to characterize signaling pathways from activated RTKs in different cell models^17–20^. In this context, receptor overexpressing cell models used in MS-based interactome studies of IR proximal signaling may lead to abnormal receptor interactions, signaling, and cellular processes^20–22^. Inducing insulin resistance in such manipulated cell line models is often complex or unachievable^20^To gain a deeper understanding of the molecular mechanisms underlying insulin resistance, we performed a multi-layered MS-based proteomics investigation in a HepG2 liver cell line model with endogenous IR expression and knock-out of the insulin-like growth factor 1 receptor (IGF1R KO). To uncover molecular changes driving insulin resistance in the model, the study focused on analyzing the insulin-dependent IR interactome, phosphoproteome, and proteome. The global phosphoproteome analysis affirmed that the insulin-resistant condition pathway specifically reduced PI3K-AKT response but not the ERK pathway. The proteome analysis revealed upregulated protein levels of the RTK, ephrin type-A receptor 2 (EphA2), in insulin-resistant compared to -sensitive cells. EphA2 belongs to the Eph receptor family and is involved in diverse cellular processes, including cell adhesion and tissue development^23,24^. Activation of the ERK pathway by insulin was responsible for upregulating EphA2 levels, and this mechanism was independent of insulin resistance. The ERK pathway-dependency was confirmed by lack of EphA2 induction upon MEK inhibition as well as by IR stimulation with the AKT-biased partial IR agonist, S597^25,26^. Finally, testing several different cell lines suggested that the ability of insulin to induce EphA2 protein levels depended on a high IR/IGF1R ratio.

## Results

To investigate the mechanisms of insulin resistance in a hepatocellular context, we employed HepG2 cells with a CRISPR-based KO of IGF1R to mimic hepatocytes of the liver, known to lack IGF1R expression^27^. Additionally, it enables a focused characterization of the IR-mediated insulin response, disregarding the influence of insulin signaling through IR-IGF1R hybrids. A cell model system of in situ hepatocyte insulin sensitivity and resistance was established using a protocol described in Dall’Agnese et al.^15^. In the applied model, HepG2 IGF1R KO cells were cultured for 48 h in serum-starvation media, followed by a 48-hour stimulation with insulin concentrations of 0.1 nM (physiological) or 3 nM (pathological) to generate insulin-sensitive and -resistant cells, respectively (Fig. S1A).

**Fig. 1:**
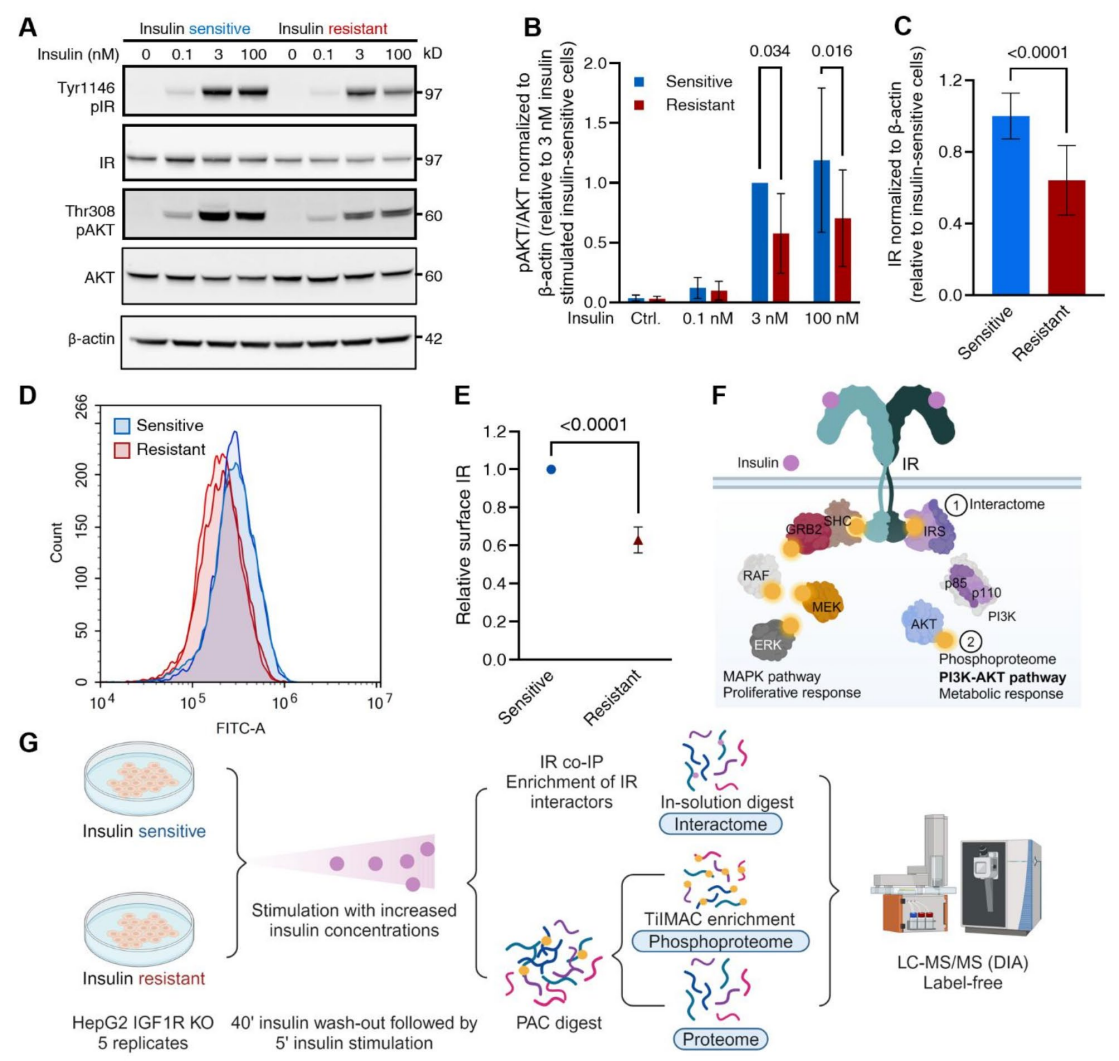
Reduced IR protein and AKT phosphorylation levels in insulin-resistant HepG2 IGF1R KO cells applied in a multi-layered proteomics experiment. (**A**) Immunoblot of insulin-sensitive and -resistant HepG2 IGF1R KO cell lysates. The cells were stimulated with increasing insulin concentrations for 5 min (representative blot of *n* = 4 independent experiments). (**B**) Phospho-AKT levels were quantified from the immunoblot shown in A and normalized to total AKT and β-actin. Insulin-sensitive and -resistant cells were stimulated with 0.1, 3, and 100 nM insulin. Unstimulated control included. Data presented as means ± SD of *n* = 3 independent experiments relative to insulin-sensitive cells stimulated with 3 nM insulin. p-values < 0.05 are indicated (2-way ANOVA). (**C**) Immunoblot quantification of IR protein levels, relative to β-actin under insulin-sensitive and -resistant conditions, independent of 5-min insulin stimulation. The data are represented as means ± SD relative to the insulin-sensitive cells (two-sample unpaired t-test) (four technical replicates from *n* = 4 independent experiments). (**D**) Flow cytometry histograms showing the average FITC signal for cell-surface IR in insulin-sensitive (blue) and -resistant (red) HepG2 IGF1R KO cells. Representative of *n* = 4 independent experiments in technical duplicates. (**E**) Quantification of surface IR levels in insulin-resistant relative to -sensitive cells, based on flow cytometry data in (**D**). (**F**) Schematic illustration of canonical insulin signaling pathways through the IR. (**G**) Workflow illustration for data-independent acquisition (DIA) MS-based interactome, phosphoproteome, and single-shot proteome analyses. Cell lysates of insulin-sensitive and -resistant cells stimulated with 0.1, 3, and 100 nM insulin for 5 min were collected. See Fig. S1E.

HepG2 IGF1R KO cells exposed to long-term pathological insulin concentrations exhibited the hallmark characteristics of insulin resistance, such as decreased levels of AKT phosphorylation upon 5-minute insulin stimulation (Fig. 1A,B). Moreover, IR protein levels were decreased by approximately 30% in insulin-resistant cells compared to insulin-sensitive cells (Fig. 1A,C, and Fig. S1B) and in agreement with this, flow cytometry confirmed reduced cell surface IR levels in the insulin-resistant cells (Fig. 1D,E). Furthermore, decreased *INSR* mRNA levels were evident in the insulin-resistant cells compared to the -sensitive (Fig. S1C) supporting a transcriptional regulation of IR expression upon resistance in this model system. These findings are consistent with other studies showing decreased IR levels in insulin-resistant states^11,12,28–30^. For the cellular response to long-term insulin treatment, the insulin-resistant cells showed approximately 33% enhanced cell viability compared to the -sensitive cells (Fig. S1D), despite their impaired insulin response involving AKT. These initial experiments confirmed that the HepG2 IGF1R KO cell model displayed the expected traits of insulin resistance, thus making it a suitable model for the analyses of hepatocellular insulin resistance.

Next, we applied a multi-layered proteomics approach to study cellular signaling alterations in insulin-sensitive and -resistant cells. The three proteomics layers analyzed consisted of the IR interactome, phosphoproteome, and proteome, sampled unstimulated or after 5-minute insulin stimulation with 0.1, 3, or 100 nM insulin (Fig. 1F,G and Fig. S1E). These datasets allowed for a comprehensive exploration of distinct aspects of insulin resistance. This study focused on two comparisons: early effects of 5-minute insulin stimulation with increasing insulin concentration in both insulin-sensitive and -resistant cells, and long-term effects of treatment with physiological or pathological insulin concentrations comparing baseline states of insulin sensitivity and resistance.

### Interactome analysis reveals distinct IR networks in insulin-sensitive and -resistant cells

**Fig. 2:**
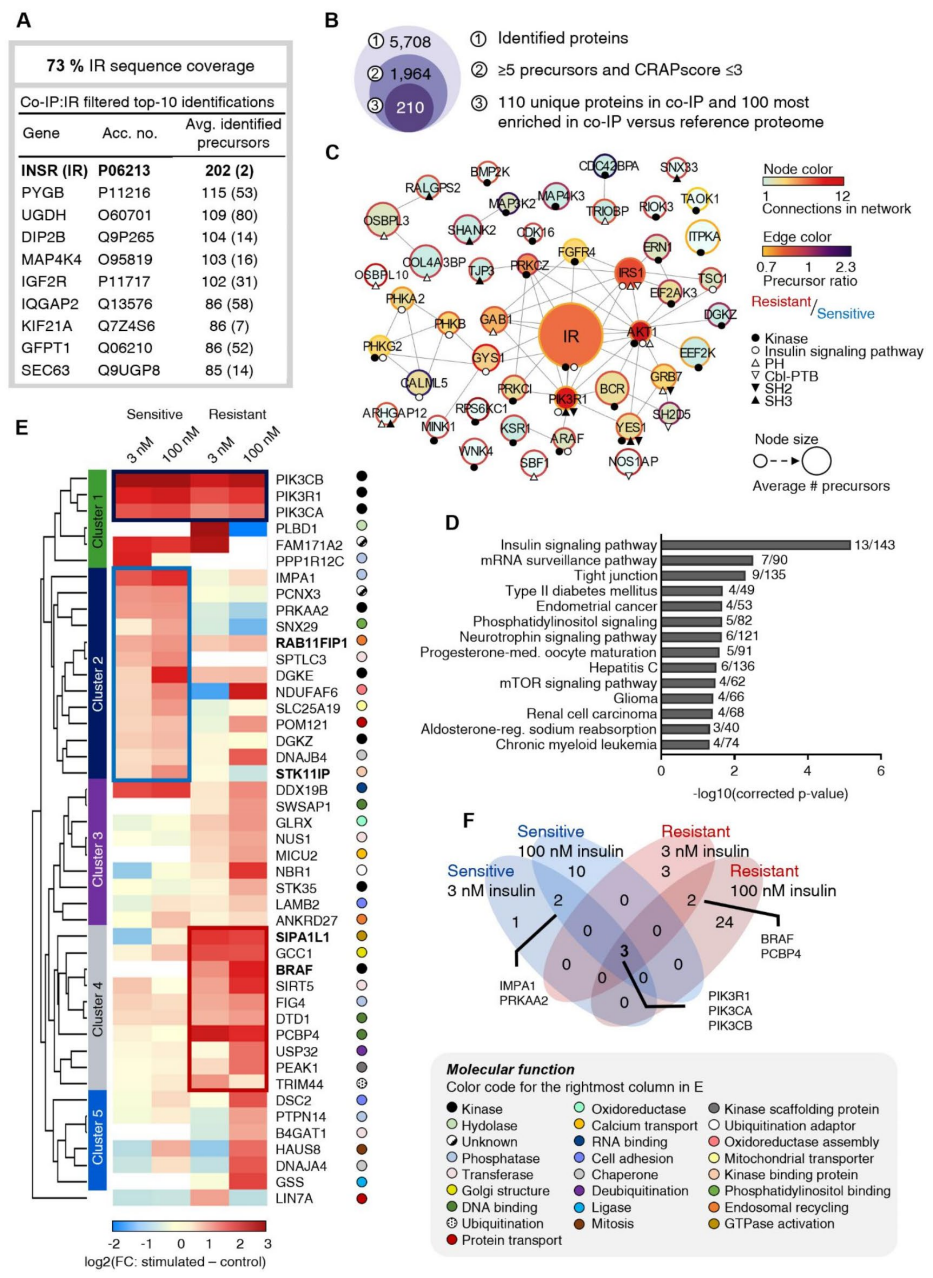
Insulin receptor interactome analysis reveals distinct signaling responses in insulin-sensitive and insulin-resistant cells. (**A**) IR sequence coverage after co-IP and listing of top 10 proteins in the co-IP dataset sorted by MS-based identified precursors, after filtration. (**B**) Number of proteins in the IR co-IP dataset after analysis filtering steps, including filtering using the Contaminant Repository for Affinity Purification (CRAPome). (**C**) Protein network of a subset of the strongest candidate IR interactors, showing kinases, proteins associated with insulin signaling, and proteins containing specific protein-protein interaction domains (SH2, SH3, PH, and Cbl-PTM domains). Node size indicates the number of annotated precursors, node color reflects connectivity within the network, and edge color represents the ratio of annotated precursors in unstimulated insulin-resistant versus sensitive conditions. (**D**) Significantly enriched KEGG pathways for candidate IR interactors (*n* = 207). (**E**) Heatmap of the filtered insulin-dependent IR interactome displaying the fold-change upon stimulation with 3 or 100 nM insulin compared to unstimulated controls in insulin-sensitive and insulin-resistant HepG2 IGF1R KO cells. Interactors significantly recruited in at least one of the conditions are included (two-sided t-test, FDR < 0.05, s0 = 0.1) (Fig. S3). Clustered based on hierarchical clustering, divided into 6 clusters (LIN7A in the bottom clustered alone). Cluster 2 (blue frame) and 4 (red frame) display a tendency for stronger interactions in insulin-sensitive and resistant cells, respectively. Colored circles, right to gene names, denote the primary molecular function of the interactors, with a specification box to the left. (**F**) Venn diagram showing the overlap of significant insulin-dependent IR interactors after stimulation with 3 or 100 nM insulin in insulin-sensitive and -resistant cells. Gene names are shown for proteins identified in more than one condition.

To study the dynamic IR interactome, a co-immunoprecipitation (co-IP) of the IR was performed, followed by label-free data-independent acquisition (DIA) liquid chromatography-tandem mass spectrometry (LC-MS/MS) analysis. A total of 5708 proteins were identified with good correlation between samples (Pearson correlation in the range of 0.63–0.95) (Fig. S2A and Table S1). To ensure a good IR enrichment in the endogenous IR-expressing HepG2 IGF1R KO cell model, IR enrichment was confirmed by immunoblot (Fig. S2B). Additionally, the IR was identified with the highest number of assigned peptide precursors (202) and a sequence coverage of 73% in the MS interactome dataset (Fig. 2A and Table S1). Furthermore, the downregulated IR levels in insulin-resistant compared to -sensitive cells were reflected in the IR levels after enrichment for the unstimulated states (Fig. S2C). These quality checks confirmed the dataset to be useful for analysis of IR signaling in a cell model with endogenous IR levels (Fig. S2A–E).

The analysis of the IR interactome involved two strategies: first, examining the core IR adaptor candidates and second, exploring the dynamic interactome, focusing on the differential regulation of IR interactors between unstimulated and insulin-stimulated conditions across the three insulin doses for insulin-sensitive and -resistant cells (Fig. S3A,B). Given that this data was based on affinity purification-MS (AP-MS) aiming to identify proteins interacting with a specific target protein, nonspecific binding comprises a general challenge. Therefore, to enhance the accuracy of potential IR binding partners, we used for filtering purposes the CRAPome (Contaminant Repository for Affinity Purification), a repository of commonly detected contaminating proteins in AP-MS studies^31^. We used inclusion criteria of ≥ 5 peptide precursors and a CRAPome score ≤ 3, shortening the IR interactome list to 1964 proteins. Based on DeepLoc prediction of subcellular localization^32^, we chose to focus on cytoplasmic adaptor proteins recruited to the intracellular part of IR. Consequently, we excluded extracellular and nuclear proteins from the analysis, resulting in a reduced list of 1310 potential interactors. To identify core components in the IR pathway, we compared the number of protein precursors assigned in the IR co-IP analysis with the reference proteome. We focused on proteins uniquely identified after the IR co-IP (110 proteins) and the 100 proteins showing the highest enrichment in the co-IP compared to the proteome (Fig. 2B). These included 25 kinases, 3 SH2 domain, 6 SH3 domain, 11 PH domain, and 3 Cbl-PTB domain proteins (Fig. 2C and Table S1). Enrichment analysis of Kyoto Encyclopedia of Genes and Genomes (KEGG)-terms confirmed the dominance of the insulin signaling pathway, supported by the identification of prominent interactors (PIK3R1, AKT, GAB1, and insulin receptor substrate (IRS)1) (Fig. 2D).

The regulation of dynamic IR interactors was characterized by significant fold-changes between the 5-min insulin stimulation and the unstimulated control, as indicated by the respective volcano plots (Fig. S3A,B and Table S1). The dynamic interactome was characterized individually for insulin-sensitive and -resistant cells to avoid biases due to the observed differences in expression levels of IR (Fig. 1A–E). The analysis showed dynamic recruitment of the PI3K complex (comprising catalytic subunits A and B, along with a regulatory subunit) in both insulin-sensitive and -resistant cells following 5 min of 3 and 100 nM insulin stimulation, but not with 0.1 nM insulin stimulation (Fig. 2E,F and Fig. S3C). This finding aligns with the absence of IR and AKT phosphorylation at the lowest insulin concentration (Fig. 1A,B).

The hierarchical clustering analysis of dynamic IR interactors divided the data into six clusters, each displaying distinct behavior between insulin-sensitive and -resistant cells. Cluster 1 revealed a consistently high level of IR-recruited PI3K complex after 5 min of stimulation with 3 and 100 nM insulin in the insulin-sensitive cells, while -resistant cells required 100 nM insulin levels for similar recruitment levels (Fig. 2E,F), thus confirming a weaker insulin responsiveness in insulin-resistant cells. Cluster 2 contained 13 interactors with increased insulin-induced recruitment in insulin-sensitive compared to resistant cells. The insulin-sensitive state interactors included rab11 family-interacting protein 1 (Rab11FIP1), which is known for its involvement in receptor tyrosine kinase recycling^17,33^. Also, 2.2-fold reduced Rab11FIP1 phosphorylation levels at Ser280 were identified in insulin-resistant cells compared to insulin-sensitive cells in the phosphoproteome dataset, following a 5-min 100 nM insulin stimulation (Table S2). Cluster 4 comprised 10 proteins showing increased IR recruitment upon 5 min of 3 and 100 nM insulin stimulation in insulin-resistant compared to sensitive cells. This cluster contained the kinase serine/threonine-protein kinase B-raf (BRAF) also demonstrating 2.7-fold increased phosphorylation levels in insulin-resistant cells at Ser602 compared to sensitive cells, following the 5-mintes stimulation with 100 nM insulin (Fig. 2E and Table S2). Cluster 5 contained 6 potential interactors showing increased recruitment for 100 nM insulin stimulation in insulin-sensitive and resistant cells, compared to their respective 3 nM stimulation. Taken together, the hierarchical clustering analysis visualized differences in the recruitment of dynamic IR interactors between insulin-sensitive and -resistant cells, which could be potential targets for further investigation into insulin resistance-associated signaling.

### Phosphoproteome analysis uncovers sustained ERK signaling and impaired insulin-stimulated AKT signaling response in insulin-resistant cells

To characterize the insulin-stimulated global phosphoproteome, a label-free quantitative DIA MS-based phosphoproteomics experiment was performed comparing control and 5-minute insulin stimulation at different doses in insulin-sensitive and -resistant cells (Fig. 1G and Fig. S1E). Among 12,688 identified and quantified phosphorylation sites, 11,398 phosphorylation sites were confidently localized with a site probability score > 0.85 and were identified in 3 ≥ samples for at least one condition (2.44% pY: 278, 19.53% pT: 2226, 78.03%, pS: 8894) (Fig. S4A and Table S2). The identified phosphorylation sites represented 2587 proteins in total (Fig. S4A). The strongest correlation (Pearson correlations 0.68–0.94) was found within the biological replicates (Fig. S4B) and median subtraction across biological replicates resulted in a stronger correlation based on insulin treatment (Fig. S4C). To gain insights into the signaling dynamics upon insulin stimulation, differentially regulated phosphorylation sites were visualized using volcano plots showing the fold change between the insulin-stimulated and unstimulated conditions against the statistical significance (t-test-based p-value) for each phosphorylation site (Fig. S4D–I). The 5-min stimulation with either 0.1, 3, or 100 nM insulin resulted in upregulated levels of 1297 and 673 phosphorylation sites in insulin-sensitive and -resistant cells, respectively (≥ 2 fold-change and *p* < 0.05) (Fig. S4J and Table S2).

**Fig. 3:**
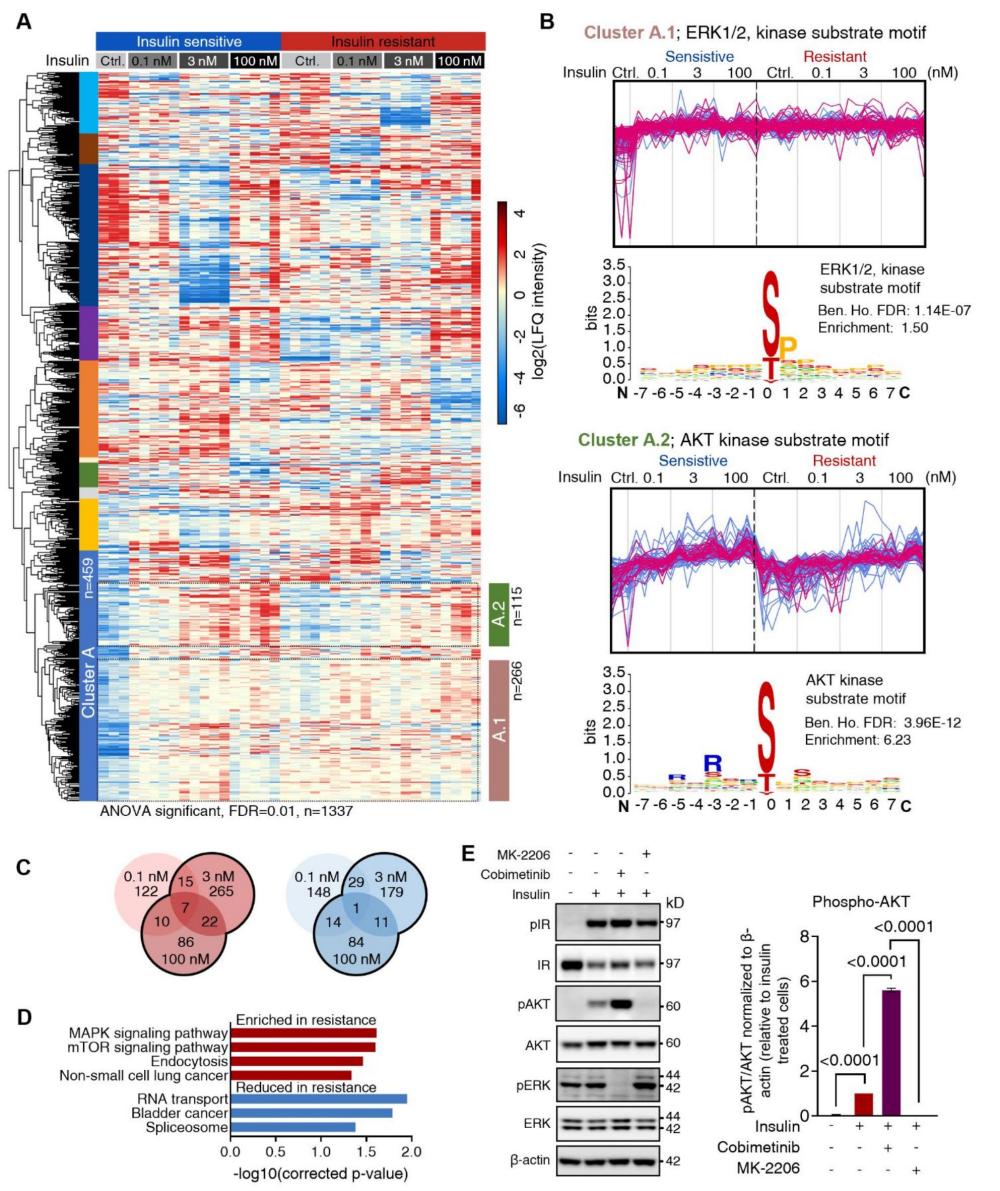
Phosphoproteomics reveals sustained ERK signaling in insulin resistance. (**A**) Hierarchical clustering of significantly regulated phospho-site levels in response to stimulation with 0.1, 3, or 100 nM insulin in insulin-sensitive and insulin-resistant cells (*n* = 1337; ANOVA; FDR < 0.01, s0 = 0.1). With 10 clusters defined from hierarchical clustering, shown as left color-bar, and subclusters of A, shown to the right. (**B**) Cluster profile and enriched sequence motif analysis for subcluster A.1 and A.2 shown in (**A**), for motifs with strongest enrichment and lowest Benjamin-Hochberg (Ben. Ho.) FDR-corrected p-values. The profiles for phosphorylation sites with ERK (top) and AKT (bottom) sequence motifs are highlighted in purple. (**C**) Venn diagram showing numbers of differentially regulated (2-fold change, *p* < 0.05) phosphorylation sites between insulin-sensitive and resistant cells. Showes sites with induced levels in insulin-resistant (red) or in sensitivity (blue) upon stimulation with 0.1, 3, or 100 nM insulin. (**D**) KEGG pathway enrichment analysis of upregulated phosphoproteins following 3 or 100 nM insulin stimulation in insulin-sensitive and -resistant cells (2-fold change, *p* < 0.05). (**E**) Immunoblot (left) and quantification (right) of HepG2 IGF1R KO cells stimulated for 24 h with 3 nM insulin without or with inhibition of AKT (MK-2206) or MEK (cobimetinib). Representative blot of *n* = 3 independent biological replicates with p-values < 0.05 annotated (two-sample unpaired t-test).

To further characterize the phosphoproteome, the level of regulation of insulin-stimulated phosphorylation sites in insulin-sensitive and -resistant cells was compared and significant regulation tested across all conditions. Here, 1337 phosphorylation sites showed significantly regulated levels and from the hierarchical clustering analysis, 10 clusters were defined (Fig. 3A). However, cluster A contained visible sub-clusters that were diverging in their phosphorylation site regulation patterns, indicating differential responses in insulin-sensitive and -resistant cells. Cluster A.1 showed decreased phosphorylation levels in unstimulated insulin-sensitive, but not in -resistant cells, compared to their respective insulin-stimulated conditions. The maximum phosphorylation site abundance was reached with 0.1 nM insulin stimulation in insulin-sensitive cells and across all conditions in insulin-resistant cells. In contrast, cluster A.2 displayed an insulin dose-response upregulation in phosphorylation levels for insulin-sensitive and -resistant cells, with a faster increase and higher maximum intensity observed in insulin-sensitive cells (Fig. 3A). A kinase-substrate motif enrichment analysis of the amino acid sequence adjacent to phosphorylation sites in cluster A.1 showed an overrepresentation of proline (P) at the + 1 position, which is the consensus motif for proline-directed kinases like ERK1/2 kinase substrates^34^. The elevated insulin concentration in the insulin-resistance-inducing protocol led to prolonged activation of ERK, which explains the sustained phosphorylation of ERK1/2 kinase substrates in the control (Fig. 3B). From an analogous analysis of cluster A.2, motifs with arginine (R) at position − 3 relative to the phosphorylated site were found to be enriched, which is the consensus motif for AKT kinase substrates^34^. This observation confirmed the reduced AKT response in insulin-resistant cells. Notably, the analysis showed differential AKT and ERK substrate motif phosphorylation response following insulin stimulation in insulin-sensitive and -resistant cells. In addition to the cluster and motif analyses, the regulated phosphorylation sites in insulin sensitivity and resistance were globally compared, focusing on high-concentration stimulations (Fig. 3C). KEGG pathway enrichment analysis further supported the upregulation of terms associated with endocytosis and MAPK pathway signaling in the insulin-resistant state (Fig. 3D).

Given the differential ERK and AKT signaling observed in insulin-sensitive and -resistant cells, the signaling response was analyzed by inhibiting these two pathways. HepG2 IGF1R KO cells were treated with 3 nM insulin alone or after pre-treatment with either cobimetinib (mitogen-activated extracellular kinase (MEK) inhibitor)^35^ or MK-2206 (AKT inhibitor)^36,37^. Immunoblot analysis confirmed that cobimetinib and MK-2206 reduced the activation of ERK and AKT, respectively (Fig. 3E). Intriguingly, inhibiting the MAPK signaling pathway led to a ≥ 5-fold increase in AKT phosphorylation, strongly suggesting crosstalk between AKT and ERK in insulin signaling, which was confirmed with an additional specific MEK inhibitor, PD0325901^38,39^, to rule out potential off-target inhibitor treatment effects (Fig. S4K–M).

### Proteome analysis reveals upregulation of EphA2 expression in insulin-resistant cells and identifies this gene as insulin-regulated

**Fig. 4:**
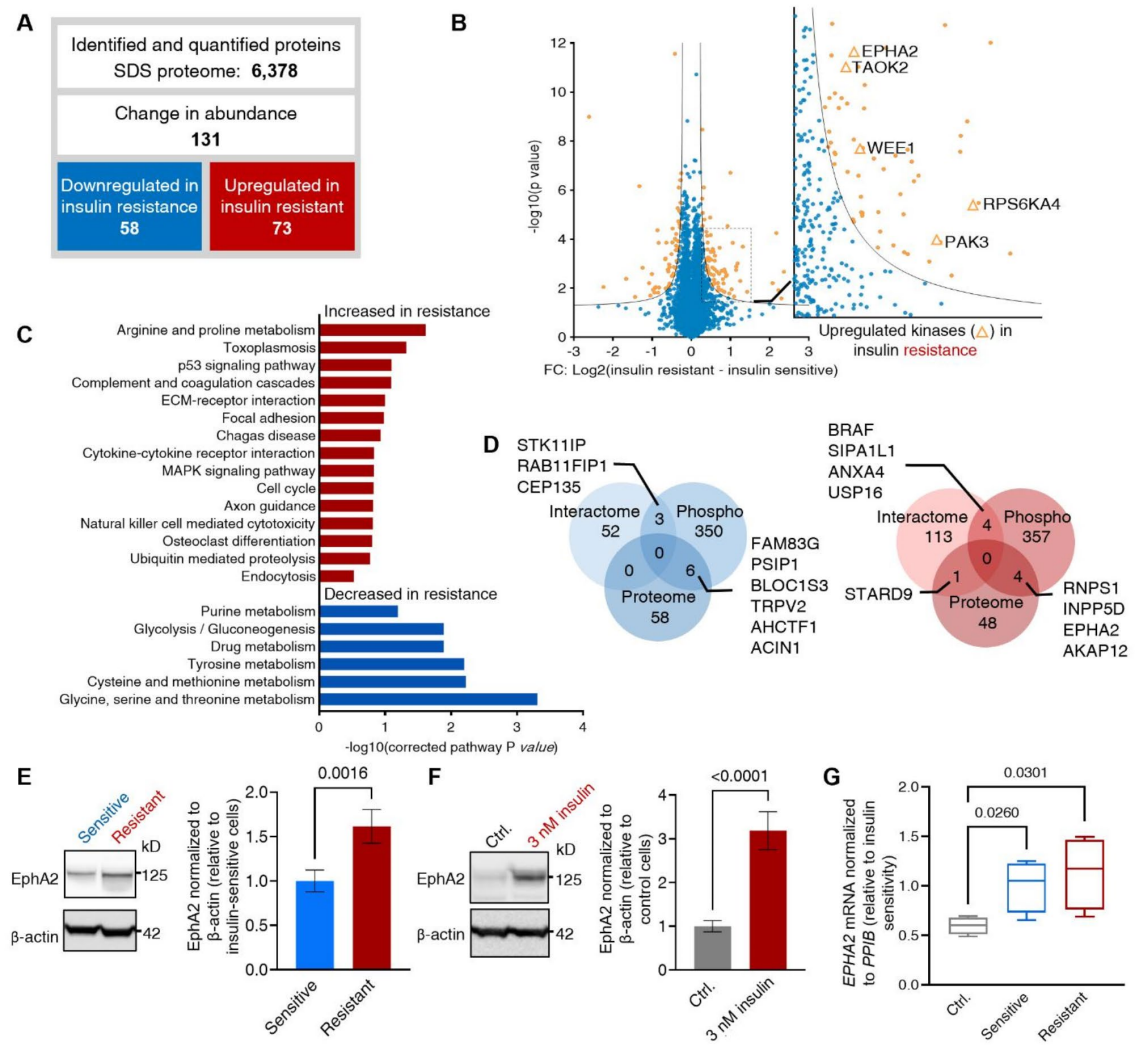
Proteome analysis shows upregulated EphA2 expression levels in insulin-resistant cells and uncovers it as an insulin-inducible gene. (**A**) Overview of proteome data: Proteins identified from the SDS-proteome dataset specifying regulated proteins in insulin-sensitive and -resistant cells. Significance based on volcano-plot in Fig. 4B made in Perseus (two-sided t-test, FDR < 0.05, s0 = 0.1). (**B**) Volcano plot presenting differentially regulated proteins in insulin-sensitive and resistant SDS-based cell lysates from single-shot proteome MS analysis. Significantly regulated proteins are highlighted in orange (FDR < 0.05, S0 = 0.1). Zoomed view shows upregulated proteins in insulin-resistant cells, with kinases labeled with gene names (*n* = 4 independent biological experiments). (**C**) KEGG term analysis of proteins significantly upregulated (red) or downregulated (blue) in insulin resistance. (**D**) Venn diagram showing overlap of upregulated proteins across the three layers of proteomics analysis. Insulin-sensitive and -resistant cells are shown in blue and red, respectively. (**E**) Immunoblot and quantification of EphA2 abundance in lysates from insulin-sensitive and -resistant cells (*n* = 4 independent biological experiments). (**F**) Immunoblot and quantification of EphA2 in unstimulated cells (48-h serum-starved control; ctrl.) or cells subjected to the insulin resistance-induction protocol (including 48-h 3 nM insulin treatment). (**G**) qPCR results of the mRNA levels of EPHA2 in HepG2 IGF1R KO cells treated as specified in (**E,F**). Median of six technical replicates for *n* = 4 biological independent replicates.

To characterize the effects of prolonged insulin treatment with 0.1 nM and 3 nM insulin during the insulin-resistance-inducing protocol on the proteome, two separate analyses were performed to identify changes in proteome abundance associated with insulin sensitivity and resistance. The first analysis served as a “reference proteome” and was based on lysates used to study the IR interactome and phosphoproteome. These cells were lysed with a milder detergent-containing lysis buffer to preserve endogenous protein-protein interactions^40^. To improve proteome coverage, a second analysis applied a harsher lysis buffer containing a highly denaturing concentration of Sodium Dodecyl Sulfate (SDS) (referred to as “SDS-proteome”)^41,42^. The reference proteome and SDS-proteome resulted in the identification and quantification of 4,867 and 6,378 proteins, respectively (Fig. S5A, Fig. 4A, and Table S3). The reference proteome displayed good correlation (Pearson correlations between 0.81 and 0.97) (Fig. S5B) between the insulin-sensitive and -resistant cells. The 5-minute insulin stimulation was assumed to have minimal effects on proteome abundance changes in insulin-sensitive and -resistant cells. The SDS-proteome dataset showed good correlation (Pearson correlation coefficients averaging 0.97) (Fig. S5D). We performed volcano plot analyses of the SDS- and reference-proteomes to identify significant fold-changes in protein levels between insulin-sensitive and -resistant cells (Fig. 4B and Fig. S5C). This analysis revealed 117 significantly regulated proteins (*p* < 0.05, ≥ 1.25-fold) for the reference proteome, (64 downregulated and 53 upregulated in insulin resistance) (Fig. S5C). In the SDS-proteome, 131 proteins exhibited significant abundance changes (FDR < 0.05, s0 = 0.1), (58 downregulated and 73 upregulated in insulin resistance) (Fig. 4A and Table S3). Among proteins with significant abundance changes, a KEGG-term enrichment analysis showed for insulin resistance an upregulation of proteins involved in MAPK signaling and axon guidance pathway and downregulation of proteins related to glycolysis, gluconeogenesis, glucose, and amino acid metabolic pathways (Fig. 4C).

Overlapping the three orthogonal datasets identified proteins differentially regulated between insulin-sensitive and -resistant cells in more than one proteomics layer (Fig. 4D). The limited number of proteins identified as regulated in the proteome while concurrently exhibiting regulation in the interactome (one in insulin-resistant) or phosphoproteome (6 in insulin-sensitive and 4 in resistant) indicated a low proteome bias in the interactome and phosphoproteome data (Fig. 4D). Proteins regulated in more than one dataset hold potential importance as they can affect various facets of cellular signaling. For example, two kinases, EphA2 and BRAF, were upregulated in two proteomics datasets in the insulin-resistant state.

In the SDS-proteome, five protein kinases were identified with upregulated protein levels in insulin-resistant compared to -sensitive cells being: EphA2, serine/threonine-protein kinase TAO2 (TAOK2), wee1-like protein kinase (WEE1), ribosomal protein S6 kinase alpha (RPS6KA), and serine/threonine-protein kinase PAK 3 (PAK3) (Fig. 4B and Table S3). Furthermore, EphA2 was confirmed to be upregulated in insulin-resistant cells from the reference proteome (Fig. S5C,E). In general, the proteins identified as commonly regulated between the two proteome analyses exhibited similar levels of fold-change regulation (Fig. S5F). The RTK EphA2 belongs to the Eph receptor family and is known to play a role in mediating cell-cell communication and regulating various cellular processes^23,24,43^. By immunoblotting, the ∼ 1.5-fold increase in EphA2 abundance at the protein level in insulin-resistant compared to insulin-sensitive cells was confirmed (Fig. 4E). To elucidate the potential role of EphA2 in insulin resistance, a small interfering RNA (siRNA) knockdown (KD) experiment, targeting EphA2 in both cell states, was performed. The immunoblot showed effective KD of EphA2 in the HepG2 IGF1R KO cell model, however, it did not restore insulin-stimulated phosphorylation levels of IR and AKT in the insulin-resistant cell model (Fig. S5G,H). Importantly, EphA2 levels were increased upon insulin stimulation, measured after 48 h, at protein and mRNA levels independently of the insulin resistance, underscoring the role of insulin signaling-dependency of these changes (Fig. 4F,G). These results confirmed an influence of chronic insulin treatment on increased EphA2 levels, and aligned with insulin resistance, as an outcome of prolonged cell culturing with high insulin concentration according to the resistance-inducing protocol. However, the regulation of EphA2 was independent of insulin resistance.

### Expression level of IGF1R correlates with regulation of EphA2 by insulin stimulation in different cell lines

**Fig. 5:**
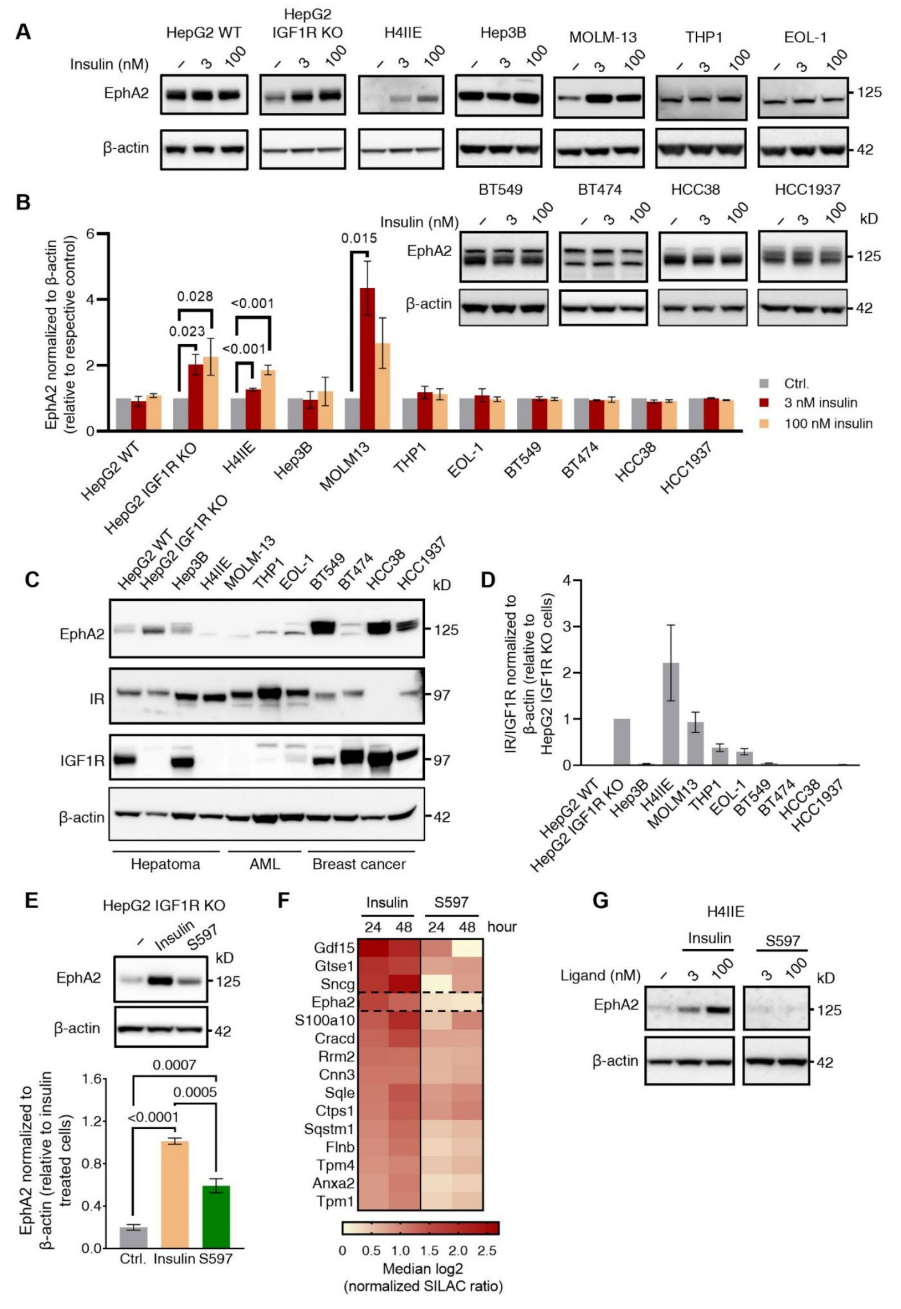
Induction of EphA2 by insulin correlates with a high IR-to-IGF1R ratio and the insulin agonist 597 does not show the same EphA2-inducing characteristics. (**A**) Immunoblot of EphA2 in a panel of screened hepatoma, breast cancer, and acute myeloid leukemia (AML) cell lines serum-starved or treated with 3 or 100 nM insulin for 24 h. Representative of *n* = 2 (*n* = 3 for H4IIE and Hep3B) biological independent experiments. (**B**) Quantification of immunoblot in (**A**). EphA2 protein levels after insulin stimulation relative to untreated (ctrl.) for individual cell lines (two-sample unpaired t-test). Normalized to β-actin levels. (**C**) Immunoblot of EphA2, IR, and IGF1R in the cell panel shown in (**A**) and (**B**). Representative of *n* = 2 biological independent replicates. (**D**) Quantification of the relative IR-to-IGF1R ratios from immunoblot in (**C**), normalized to β-actin levels. (**E**) Immunoblot and quantification of EphA2 in HepG2 IGF1R KO cells untreated or treated with 100 nM insulin or the partial IR agonist S597 for 24-hours (two-sample unpaired t-test) (*n* = 4 independent biological experiments). (**F**) Heatmap of median log2 protein SILAC ratios for significantly insulin-induced protein levels in H4IIE cells (significance B testing, FDR < 0.05). Cells were cultured with 100 nM insulin or S597 for 24–48 h (*n* = 3 independent biological experiments). (**G**) Representative immunoblot of EphA2 in H4IIE cells treated for 24 h with 100 nM insulin or S597 (two-sample unpaired t-test) (*n* = 3 independent biological replicates).

Since EphA2 KD did not restore AKT signaling in insulin-resistant cells to the level in -sensitive cells, we assumed that the upregulation of EphA2 did not play a causal role in resistance. However, we speculated whether the insulin dependency of EphA2 expression was a general finding across other cell types and therefore, HepG2 WT cells were treated with 3 and 100 nM insulin for 24 h (Fig. 5A,B). Unlike in HepG2 IGF1R KO cells, insulin stimulation did not induce the expression of EphA2 in HepG2 WT cells, indicating an essential role of IGF1R. Therefore, 11 insulin-responsive cell lines were screened for insulin-induced EphA2 regulation, including four hepatoma (HepG2 WT, HepG2 IGF1R KO, Hep3B, and H4IIE), three acute myeloid leukemia (AML) (MOLM-13, THP1, and EOL-1), and four breast cancer cell lines (BT549, BT474, HCC38, and HCC1937). The IR-to-IGF1R ratio was generally higher in hepatic and AML cells, while breast cancer cells showed varying but lower ratios given higher IGF1R expression levels (Fig. 5C,D). For the hepatoma cell lines, insulin stimulation induced EphA2 expression in HepG2 IGF1R KO and the rat hepatoma H4IIE cells known to lack IGFIR expression without manipulation^44^, but not in HepG2 WT and Hep3B cell lines. Among the AML cell lines, an ~ 5-fold increase of EphA2 protein level with 3 nM insulin stimulation was observed for MOLM-13, whereas THP1 and EOL-1 cells showed no EphA2 induction. None of the screened breast cancer cell lines showed EphA2 protein regulation after insulin stimulation (Fig. 5A,B). HepG2 IGF1R KO, H4IIE, and MOLM-13 cells exhibited the lowest IGF1R protein levels, supporting the hypothesis of a requirement for low or absent IGF1R expression for insulin-induced EphA2 upregulation (Fig. 5C,D).

Since the phosphoproteome of the insulin-resistant cells showed sustained ERK signaling, we speculated whether the balance between PI3K-AKT and MAPK downstream signaling impacted the regulation of EphA2. Therefore, HepG2 IGF1R KO cells were stimulated for 24 h with 3 nM and 100 nM insulin or the partial IR agonist S597, which has previously been shown to primarily signal via the PI3K-AKT pathway^25^. Intriguingly, stimulation with S597 did not result in upregulated EphA2 expression at the protein level, compared to stimulation with insulin. This supported the dependency of induced ERK signaling, in the insulin-induced upregulation of EphA2 (Fig. 5E). To confirm the observed regulation of EphA2 protein levels by insulin but not S597 in another hepatic cell model, the rat hepatoma cell line H4IIE was used. The changes in protein abundance following stimulation with insulin and S597 were additionally explored with a stable isotope labeling by amino acids in cell culture (SILAC) experiment in H4IIE cells. The SILAC-labelled cells were stimulated with 100 nM insulin or S597 for 24 and 48 h followed by an MS proteome analysis (Fig. S6A,B). In total 7508 proteins were identified and quantified and EphA2 protein levels were upregulated after 24 and 48 h of insulin stimulation, ranking EphA2 as the fourth most insulin-induced protein. In contrast, S597-stimulation did not lead to detectable upregulation of EphA2 protein levels, verified by immunoblotting (Fig. 5F,G). This supported our hypothesis that insulin regulates EphA2 induction via activation of the MAPK signaling pathway.

### MAPK signaling drives insulin-induced EphA2 expression in cell models with a high IR/IGF1R ratio

**Fig. 6:**
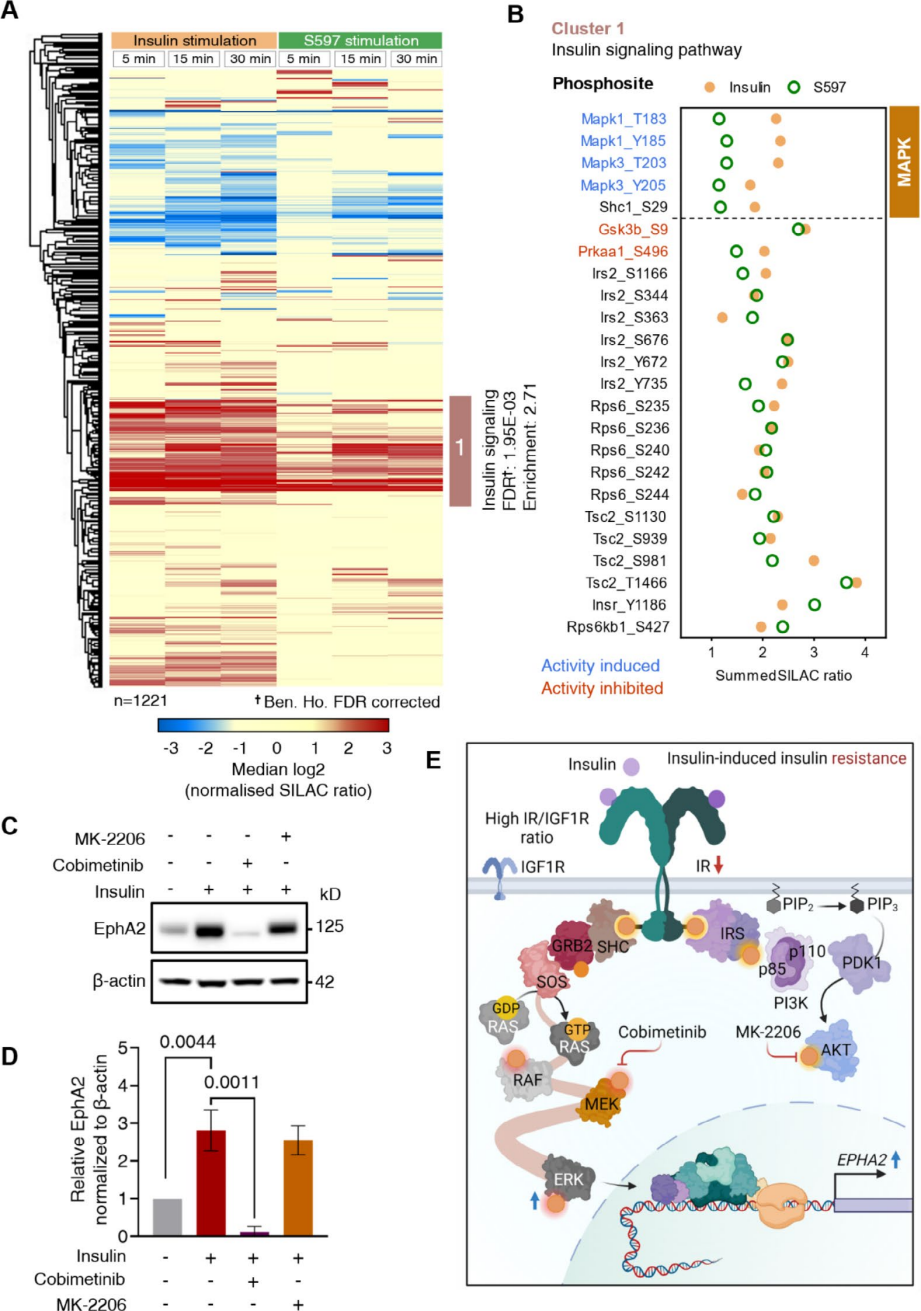
SILAC phosphoproteomics validates primary AKT-directed response by S597 and reveals insulin-mediated EphA2 expression via the MAPK pathway. (**A**) Heatmap displaying phosphorylation site SILAC ratios regulated ≥ 2-fold after 5-, 15-, or 30-min stimulation with 100 nM insulin or S597 in H4IIE, in at least one condition. The cluster with the overrepresented KEGG insulin signaling pathway is marked. Median SILAC ratios from *n* = 2 independent experiments. (**B**) Plot illustrating KEGG insulin signaling pathway phosphorylation sites (labeled cluster in **A**). Median SILAC ratio, averaged for all time points for insulin (solid yellow) and S597 (hollow green). Regulatory sites are color-coded based on whether phosphorylation induces (blue) or inhibits (red) protein activity. (**C**) Immunoblot of HepG2 IGF1R KO lysates treated for 24 h with 1 µM MK-2206 (AKT) inhibitor, 1 µM cobimetinib (MEK) inhibitor, and 3 nM insulin. Immunoblots of pIR, IR, pAKT, AKT, pERK, and ERK are shown in Fig. 3E. (**D**) Quantification of EphA2 protein levels, based on immunoblot in (**C**). Normalized to β-actin levels. (**E**) Schematics depicting dysregulation of MAPK signaling in insulin resistance showing the connection to induced EphA2 expression levels and IR/IGF1R ratio dependence.

To validate S597 signaling primarily through the PI3K-AKT pathway and elucidate potential differences in downstream signaling by insulin and S597, a SILAC-based phosphoproteome analysis was performed in H4IIE cells. The cells were stimulated with 100 nM insulin or S597 for 5, 15, or 30 min before lysis and MS analysis. We identified 11,995 confidently localized phosphorylation sites (class I) showing a strong correlation across the different conditions (Fig. S6C,D and Table S4). A heatmap of phosphorylation sites with differentially regulated SILAC ratios (≥ 2-fold in at least one condition), highlighted an enriched cluster associated with the insulin signaling pathway (Fig. 6A). In cluster 1, representing phosphorylation sites within proteins associated with the insulin-signaling pathway, phosphorylation linked to the AKT-mediated insulin response exhibited similar patterns for both ligands as shown by Jensen et al.^27^. S597-stimulated cells displayed more pronounced IR Tyr1186 phosphorylation, confirming IR activation by S597 (Fig. 6B). Furthermore, elevated levels of MAPK phosphorylation (ERK2 Thr183 and Tyr185; ERK1 Thr203 and Tyr205) were observed following insulin stimulation, which was less pronounced with S597, aligning with the previous findings on S597-mediated signaling^25^ (Fig. 6B).

The reduced ERK signaling and decreased EphA2 induction upon stimulation with S597, compared to insulin stimulation, supported the hypothesis of MAPK-mediated EphA2 regulation by insulin in the IGF1R-deficient cell line models. To further test this hypothesis, EphA2 expression in HepG2 IGF1R KO cells treated with insulin alone or after pre-treatment with either the MEK inhibitor cobimetinib or the AKT inhibitor MK-2206 were examined, using the same lysates employed to evaluate the interaction between AKT and ERK phosphorylation levels in insulin signaling (Fig. 3E). Immunoblot data confirmed a lack of insulin-induced EphA2 expression in cells subjected to MEK inhibition, in contrast to cells treated with insulin alone or in combination with AKT inhibitor (Fig. 6C,D). This confirmed that induction of EphA2 gene expression by insulin is dependent on MAPK pathway activity in this IGF1R-deficient cell model (Fig. 6E).

## Discussion

In this study, we employed a multi-layered proteomics approach to characterize insulin signaling under insulin-sensitive and -resistant conditions in a hepatocellular context. We adapted the insulin resistance-inducing protocol established by Dall’Agnese et al.^15^. but utilized HepG2 IGF1R KO cells, which as healthy hepatocytes do not express IGF1R^27^, in contrast to the HepG2 WT. Although IR and IGF1R share significant homology and engage similar signaling pathways, IR generally regulates metabolic processes, while IGF1R primarily controls growth^45^. The HepG2 IGF1R KO and WT cell models exhibited decreased metabolic AKT signaling during insulin resistance. However, differences were observed in other aspects, including the regulation of IR levels, the ERK signaling response, and insulin-induced EphA2 levels.

In the insulin-resistant condition, mRNA, total protein, and surface IR levels were decreased in HepG2 IGF1R KO cells, which differed from the results in HepG2 WT cells^15^. Despite inconsistent findings, studies indicate that chronically elevated insulin levels can result in reduced levels of IR in insulin-resistant and diabetic systems^11–13,28^ and hence, justifies our model system. In the IR interactome analysis, we identified the Rab11FIP1 protein, which is implicated in intracellular vesicle trafficking and RTK recycling^17,33^ making it interesting for further validation. It is also reasonable to speculate that the observed increased basal ERK signaling may link to elevated IR endocytosis, reducing surface IR expression and facilitating the development of insulin resistance^46–49^. Compared to previous interactome studies primarily using IR overexpression cell models, we identified 45 dynamic IR interactors, which is deemed a modest number of IR interactors, however likely a natural consequence of studying an endogenous expression system^18,20–22^.

The HepG2 IGF1R KO cells showed sustained ERK activation, potentially suppressing further activation by 5-minute insulin stimulation. The increased viability in insulin-resistant cells compared to the insulin-sensitive cells is most likely supported by the increased basal ERK signaling. The phenomenon of insulin resistance leading to a higher basal ERK signaling state is commonly observed^50^. Inhibition of the MAPK-ERK signaling pathway in HepG2 IGF1R KO cells resulted in an increased PI3K-AKT activation by insulin, suggesting a cross-talk mechanism between ERK and AKT pathway activation following insulin stimulation. Others have investigated crosstalk between AKT and ERK signaling pathways, including in insulin resistance contexts^51–53^. Interestingly, a study showed improved insulin sensitivity and glucose tolerance in diabetic mice treated with a MEK inhibitor^47^, supporting the need to explore IR agonists like S597, known for biased AKT signaling^25^, in insulin resistance research.

A key finding in our study was the upregulation of EphA2 protein levels by insulin through the ERK signaling pathway. However, whereas other studies confirm the link between active ERK signaling and increased EphA2 expression^54–58^, our study is the first to demonstrate that insulin-stimulated EphA2 induction was specific to cells with a high IR-to-IGF1R ratio. EphA2 is involved in cell adhesion, migration, proliferation, and tissue development. However, the implications of increased EphA2 levels in the HepG2 IGF1R KO cell model with induced insulin resistance remain unknown. Another receptor from the Eph receptor family, ephrin type-B receptor 4 (EphB4), has been shown to interact with and drive IR degradation after insulin stimulation^30^. Based on our interactome analysis, an interaction between IR and EphA2 was not observed, and a similar role to that observed for EphB4 thus cannot be confirmed. However, further experiments are needed to confirm this.

The evidence of a high IR-to-IGFR ratio in the insulin-induced upregulation of EphA2 suggests the potential involvement of IGF1R levels in insulin signaling. The ability of IR to form hybrid receptors with IGF1R in cells co-expressing the two receptors adds an intriguing dimension to the already complex interplay of the two receptors. Studies have shown that hybrid receptors have reduced insulin affinity compared to the IR and have been associated with metabolic and mitogenic dysregulated diseases, such as insulin resistance and certain types of cancer^59–63^. It is reasonable to speculate that hybrid receptors could contribute to changed ERK signaling linked to the increased insulin-induced EphA2 expression.

Our study investigated insulin signaling in insulin resistance using a liver cell model undergoing an insulin resistance-inducing protocol. Along these lines, enhancing the research with primary cells or animal models would strengthen the physiological relevance of our findings and add to strengthen our conclusions. The IR interactome analysis revealed a limited number of dynamic IR interactors in the interactome dataset as a likely consequence of analysis of endogenous IR levels. Thus, improved analysis sensitivity through method optimization could improve interactor identification. For instance, the available proximity labeling systems offer robust methods for identification of also weak and transient interactors to fully comprehend the network of IR interactions that influence insulin resistance. However, these engineered systems often challenge cell models with artificial overexpression of fusion constructs. Knowing that signaling events are intrinsically coordinated in time and space, our analysis of the IR interactome and phosphoproteome merely represent snapshots of a given time-point at three different insulin concentrations. Therefore, future studies applying a spatio-temporal approach to insulin signaling are essential to fully capture and characterize the extended network dynamics to identify differences upon insulin sensitivity and resistance.

In conclusion, this multi-layered proteomics study serves as a comprehensive resource, covering the IR proximal and downstream signaling dynamics as well as global proteome changes in an insulin-sensitive and -resistant hepatocellular cell line model with endogenous IR levels and a KO of IGF1R. Using this integrative proteomics approach, we highlighted the role of ERK signaling on the proteome landscape and found that insulin, independently of insulin resistance, induces ERK-dependent EphA2 expression exclusively in cells with a high IR-to-IGF1R ratio.

## Methods

### Reagents

Human insulin (Novo Nordisk A/S) and S597^26^ (Novo Nordisk A/S) was utilized for the experiments. The following antibodies were applied for Western blots (WBs): rabbit anti–phospho–IGF-1Rβ (Tyr1131)/insulin receptor β (Tyr1146), rabbit anti–insulin receptor β (4B8), rabbit IGF-I Receptor β (111A9), rabbit anti–phospho-Akt (Thr308) and anti-Akt, mouse anti–phospho-ERK1/2 (Thr202/Tyr204), rabbit anti-ERK1/2 (Cell Signaling Technology); rabbit anti-Eph receptor A2 antibody (ab273118 and ab185156), mouse anti-beta actin antibody (ab8226) (Abcam); insulin receptor β antibody (CT-3) (Santa Cruz); goat anti-rabbit immunoglobulin (IgG) secondary antibody, horseradish peroxidase (HRP)–conjugated, and goat anti-mouse IgG secondary antibody, HRP-conjugated (Bio-Rad). For phospho (p)Tyr enrichment in the SILAC experiment, pTyr1000 (8803) and pTyr100 (5636) (Cell Signaling Technology) were used. For the co-IP, the mouse-anti insulin receptor (83 − 7) obtained under license from Professor K. Siddle, University of Cambridge, UK^64^ was used. For flow cytometry analysis, the following antibodies were used: anti IR (D2) mouse IgG110^65^ and mouse IgG1 Neg control (DAKO). The kinase inhibitors used were cobimetinib (S8041), PD032591 (S1036), and MK-2206 (S1078) (Selleckchem).

### Maintenance cell culture

The human hepatoma cell lines: HepG2 IGF1R KO (in-house CRISPR KO), HepG2 WT (HB8065, ATCC), and Hep3B (HB8064, ATCC) were cultured in DMEM, 4.5 g/L D-glucose (Gibco). The human breast cancer cell lines; BT549 (HTB-122, ATCC), BT474 (HTB-20, ATCC), HCC38 (CRL-2314, ATCC), and HCC1937 (CRL-2336, ATCC) were cultured in RPMI 1640 (Gibco), all supplemented with 10% fetal bovine serum (FBS) and penicillin (100 U/mL) and streptomycin (100 µg/mL) (P/S) (Gibco). The rat hepatoma cell line, H4IIE (CRL-1548, ATCC), was cultured in MEM media (Gibco) with 1% MEM Non-Essential Amino Acids Solution (Gibco) and 1% pyruvate (Gibco) and supplemented with 10% FBS and P/S. The human AML cell lines MOLM-13 (ACC-554, DSMZ), THP-1 (TIB-202, ATCC), and EOL-1 (ACC-386, DSMZ) were cultured in RPMI with Glutamax (Gibco), supplemented with 10% FBS and P/S. Additionally, for the THP-1 cells, 20 nM 4-(2-hydroxyethyl)-1-piperazineethanesulfonic acid (HEPES) (Gibco) and 50 µM 2-Mercaptoethanol (Gibco) were added, while 20 nM glutamine (Gibco) were added for the EOL-1 cells. The HepG2 IGF1R KO and HepG2 WT cells were cultivated on collagen-coated surfaces.

### IGF1R KO in HepG2

The HepG2 IGF1R KO cell line was generated by transfecting HepG2 WT with Cas9 protein, crRNA targeting exon1, and tracrRNA in IDTE buffer (Integrated DNA Technologies) using Neon transfection kit (Invitrogen). The transfected cell pool was expanded from 24 W plate to T75 flask. Using human IGF1R PE-conjugated antibody (R&D systems), the IGF1R negatively stained population was bulk-sorted using SH800 cell sorter (Sony) to select for IGF1R KO cells. After expansion, the cell pool was limited diluted into Nunclon delta surface 96 W plates (Thermo Scientific) using medium supplemented with 50% conditioned medium. Surviving clones were expanded in poly-D-lysine coated plates (Corning) and screened with Zero Blunt TOPO PCR cloning (Invitrogen) and Sanger sequencing (Eurofins), leading to the selection of the HepG2 IGF1R KO clone.

### Cell treatments

To mimic insulin sensitivity and resistance in HepG2 IGF1R KO cells, cells were seeded in DMEM 1 g/L D-glucose (Gibco) with 10% FBS and 1% P/S. The following day media was exchanged to DMEM, 1 g/L D-glucose with 1% P/S for 48 h (serum washout). Next, media was exchanged to DMEM, 1 g/L D-glucose with 1.25% human serum albumin (HSA) (Sigma) and either physiologic (0.1 nM) or pathologic (3 nM) insulin for 48 h. The insulin-sensitivity and -resistance protocol was adapted from Dall’Agnese et al.^15^.

For experiments on the cell line panel, cells were seeded in their respective growth media and serum-starved overnight in media without FBS. After serum washout, cells were either continued serum-starved or treated with 3 or 100 nM insulin with 1.25% HSA for 24 h.

In the ERK and AKT inhibition experiment, HepG2 IGF1R KO cells were serum-starved overnight in DMEM 1 g/L D-glucose with 1% P/S. Next pretreated with cobimetinib or PD032591 (MEK inhibitors) or MK-2206 (AKT inhibitor) for 30 min, followed by 24 h co-treatment with 3 nM insulin.

### Cell stimulation and lysis

To study IR activation in insulin-sensitive and -resistant HepG2 IGF1R KO cells, an extensive seven-step insulin wash-out was performed with DMEM, 1 g/L D-glucose media. The procedure comprised three continual media exchanges followed by three 5-min exchanges, and one 20-min exchange, with incubation in a cell incubator for the 5- and 20-min steps.

After the insulin wash-out, cells were stimulated for 5 min with either 0, 0.1, 3, or 100 nM insulin in DMEM, 1 g/L D-glucose with 1.25% HSA in a cell incubator. Cells were washed and lysed directly in experiments without specified insulin stimulation. Wash was performed with phosphate-buffered saline (PBS) and lysis with co-IP lysis buffer [50 mM tris-HCl (pH 7.5), 150 mM NaCl, 1 mM calcium chloride, 1% Triton X-100] added 5 mM β-glycerophosphate, 5 mM sodium fluoride, 1 mM sodium ortho-vanadate, and one cOmplete EDTA-free Protease inhibitor tablet (Roche) per 10 mL solution. For SDS-proteome analysis, lysates were collected after a 48-h differential insulin treatment with 0.1 nM (insulin-sensitive condition) or 3 nM insulin (insulin-resistant condition), without 5-minute insulin stimulation. Cell pellets were washed twice with PBS and lysed by heating at 95 °C for 10 min in lysis buffer consisting of 100 mM Tris-HCl (pH 8.5), 5% SDS, 5 mM TCEP, and 10 mM CAA. Sonication was performed thereafter. For all samples protein concentration was determined using BCA Protein Assay Kit (Pierce).

For the quantitative SILAC MS-based proteomics and phosphoproteomics experiments, H4IIE cells were subjected to SILAC for a minimum of 14 days. The SILAC media was composed of MEM for SILAC (Thermo Scientific), 1% P/S, 10% dialyzed FBS for SILAC (Gibco), 1% MEM Non-Essential Amino Acids Solution, sodium pyruvate, and GlutaMAX (Gibco). Three different cell populations were achieved by the addition of 166 µM lysine (Lys) and 274 µM arginine (Arg) in the following variations: Light: L-Arg0 and L-Lys0 (Sigma) Medium: Arg6 (L-(13C6) Arg) and Lys4 (L-(2H4) Lys). Heavy: Arg10 (L-(13C6, 15N4) Arg) and Lys8 (L-(13C6, 15N2) Lys). The medium and heavy labeled amino acids were obtained from Cambridge Isotope Laboratories (Tewksbury).

For phosphoproteome analysis, SILAC H4IIE cells were serum-starved overnight in SILAC media containing 0.1% FBS, before 100 nM insulin, 100 nM S597, or vehicle was added for 5, 15, or 30 min. At the end of stimulation, cells were washed five times in ice-cold DPBS and lysed in 6 M guanidine hydrochloride (Sigma-Aldrich) in 10 mM Tris, pH 8, added PhosSTOP (Roche). Protein concentrations were measured by BCA. For one biological replicate, 2 mg protein from each treatment condition was mixed 1:1:1, to give a final protein content of 6 mg and for the second replicate, a 1:1:1 mixture was composed of 4 mg from each condition.

For proteome analysis, SILAC H4IIE cells were treated for 24–48 h with regular SILAC media containing 100 nM human insulin, 100 nM S597, or vehicle. For the 48 h stimulation, the media was changed after 24 h of stimulation. Following treatment, the cells were washed five times in ice-cold DPBS and lysed with 0.5% RapiGest (Waters) in 50 mM triethylammonium bicarbonate (TEAB) buffer. Cell lysates were collected and 1 µL Benzonase nuclease (EMD Chemicals) was added. Protein concentrations were measured by BCA. Finally, 200 µg proteins from each treatment condition were mixed giving a 1:1:1 protein mixture ready for digestion.

### Cell viability assay

HepG2 IGF1R KO cells were seeded in black/clear bottom 96-well cell culture plates (Fisher Scientific) and subjected to the insulin resistance induction protocol. As described, after 48-h serum-starvation, cells were cultured with 0.1 or 3 nM insulin in DMEM, 1 g/L D-glucose with 1.25% HSA for 48 h. Cell viability was assessed using the CellTiter-Glo^®^ Luminescent Cell Viability Assay (Promega) following the manufacturer’s instructions. Luminescence was measured at 37 °C using a CLARIOstar^®^ plate reader.

### Assay for insulin receptor and AKT activation

Cells were seeded in 96-well cell culture microplates (Fisher Scientific) and subjected to the insulin-resistant protocol as described in the previous section. A seven-step insulin washed-out was performed before 5-minute insulin stimulation with doses ranging from 0 to 600 nM. Reagents were supplied in the AlphaScreen, SureFire, Insulin Receptor (p-Tyr1150/1151), and AKT 1/2/3 (p-S473) Assay Kits (PerkinElmer). Cell lysis and SureFire Assay were performed following the manufacturer’s instructions.

### siRNA experiments

HepG2 IGF1R KO cells were transfected in 6-well cell culture plates (Fisher Scientific) using Lipofectamine RNAiMAX Transfection Reagent (Thermo Fisher Scientific). Following 48-h serum-free incubation, the cells were treated with the transfection reagent in DMEM, 1 g/L D-glucose, supplemented with 1.25% HSA and either 0.1 nM or 3 nM insulin for 12 h. The EphA2 siRNA SMARTpool ID: L-003116-00-0005 (Dharmacon Inc.) or the ON-TARGETplus Non-targeting Control SMARTpool ID: D-001810-10-05 (Dharmacon Inc.) was added to a final concentration of 48 nM. After 12 h of transfection, the medium was exchanged and the cells were cultured with insulin for an additional 36 h, for 48 h of incubation, following the insulin resistance-inducing protocol. Subsequently, the insulin was washed-out, and the cells were stimulated with insulin as previously specified before being lysed for WB analysis.

### Western blotting

SDS-PAGE (4–12% Bis-Tris, MOPS buffer, NuPAGE, Invitrogen) was blotted to nitrocellulose transfer membranes according to the manufacturer’s protocol (iBlot). SuperSignal West Dura Extended Duration Substrate or West Pico Chemiluminescent Substrate (Thermo Scientific) was used for detection with an LAS-3000 Imaging System (Fuji). Reprobing of blots after restoring Western Blot Stripping Buffer (Thermo Scientific). Quantitative analysis of bands of interest was done using either Image Gauge v.4.0 or ImageJ software tools.

### Flow cytometry

Cell surface IR receptor levels were quantified using the QIFIKIT kit (DAKO) for flow cytometry analysis. HepG2 IGF1R KO cells were cultured in 6-well plates and detached with Versene (Gibco) for 5 min at 37 °C. The cells were washed and resuspended in cold FBS Stain Buffer (BD Biosciences). 2 × 10^4^ cells per condition were stained with anti-IR (D2) mouse IgG1 or isotype control and incubated in the dark for 1 h at 4°C followed by three washing steps in cold FBS Stain Buffer. Subsequently, the cells were incubated at room temperature in the dark for 45 min with anti-mouse secondary antibodies conjugated to Alexa Fluor (FITC). After three washing steps, the cells were resuspended in 200 µL FBS Stain Buffer for flow cytometric analysis.

Flow cytometry data was acquired using a NovoCyte Quanteon flow cytometer, recording 15,000 events of the desired and gated populations. The instrument was operated with a sample volume of 75–100 µL, a flow rate of 35 µL/sec, and the FITC channel set at 480 nm for fluorescence detection. Data analysis was performed using NovoExpress v.1.5.6 software, and single-cell populations were gated based on a plot of side scatter (SSC-A) versus forward scatter (FSC-A) light signals.

### Real-time PCR and RNA sequencing

RNA was extracted after 24-h culturing in DMEM, 1 g/L D-glucose, 10% FBS, with 1% P/S, after 48 h in DMEM, 1 g/L D-glucose with 1% P/S, and after inducing insulin sensitivity and resistance according to the protocol described above. RNA extraction was performed using the RNAdvance Cell v2 Kit (Beckman Coulter). The RNA concentration was determined using NanoDrop™ 8000, and 300 ng was used as starting material for cDNA synthesis, applying the iScript cDNA Synthesis Kit (Bio-Rad). Real-time PCR was performed on a Thermo Fisher Scientific QuantStudio 12 K Flex using TaqMan™ Fast Advanced Master Mix (Thermo Fisher). TaqMan primers (Thermo Fisher) for the IR, EphA2, and PPIB genes were used with the following IDs: INSR: Hs00961554_m1, EPHA2: Hs01072272_m1, PPIB: Hs00168719_m1. For each condition, the average of 12 technical replicates was used as the representative Ct value of each biological replicate (*n* = 4). The Ct values were normalized by determining the expression levels of the target genes relative to the endogenous housekeeping gene PPIB. To correct the expression of the target gene relative to PPIB, the ΔCt method was applied, where ΔCt is the difference in Ct values between the target gene and PPIB. The relative quantification (RQ) was calculated using the formula RQ = 2^−ΔCt^^66^. The corrected individual Ct values were normalized to the average of the biological replicates for the chosen reference condition.

### Sample preparation for mass spectrometry-based proteomics

For co-IP with the IR for MS-based IR interactome analysis, the IR-specific 83 − 7 antibody was coupled to Dynabeads™ MyOne™ Tosylactivated magnetic beads (Invitrogen™) following the manufacturer’s instructions. Non-conjugated beads were prepared in parallel, with no addition of antibody in the conjugation step. 500 µg of protein was used for IR co-IP. The lysate was diluted to a concentration of 0.5 mg/mL in IP-bead incubation buffer [50 mM HEPES; 125 mM NaCl; 0.5 mM CaCl_2_; 5 mM MgSO_4_; 0.0125% Tween20; 0.5% TritonX100] and 100 µg of non-conjugated beads were added to each sample for pre-clearing. This was done in a 96-deep well format, with samples incubated shaking at 750 rpm at 4 °C for 1 h. While keeping samples cold, the supernatant was carefully transferred to a new 96-well plate to prevent any bead contamination. Afterward, 150 µg of antibody-conjugated beads were added to each sample, followed by incubation while shaking at 750 rpm at 4 °C for 4 h. After the IR co-IP, the beads were passed through an automated washing procedure on a KingFisher robot with five 1-minute washing steps in the IP-bead incubation buffer. Finally, the protein was eluted using 100 µL of IgG Elution buffer (Pierce™).

For MS-based single-shot proteome and phosphoproteome analysis, each sample was digested and then split for either direct single-shot proteome or phosphopeptide enrichment. A total of 500 µg of protein from the co-IP lysate and the SDS-lysate were separately digested overnight at 37 °C on a Kingfisher Flex robot using the protein aggregation capture (PAC) method^38,41^. The digestion was performed in a 50 mM TEAB buffer with 0.2 µg of Lys-C (Wako Chemicals) and 0.4 µg of trypsin (Promega). Trifluoroacetic acid (TFA) acidification was applied to quench the digestion, followed by loading of 750 ng of peptides onto Evotip Pure (Evosep) disposable trap columns for single-shot proteome analysis of both lysates. The remaining peptides from the digested co-IP lysate, corresponding to 200 µg peptide mixtures, underwent clean-up using SepPak (C_18_ Classic Cartridge, Waters). Elution was performed using 40% and 60% acetonitrile (ACN). The samples were then lyophilized, resuspended in a loading buffer composed of 80% ACN, 5% TFA, and 1 M glycolic acid, and subjected to automated Ti-IMAC (titanium immobilized metal affinity chromatography) phosphopeptide enrichment^38,67^. TiIMAC-HP beads (MagReSyn, Resyn Biosciences) were incubated with the peptides for 20 min, followed by successive washing steps with the loading buffer, 80% ACN, 1% TFA, and 10% ACN, 0.2% TFA. Phosphopeptides were eluted in 1% ammonia and clarified by filtration using a 0.45 μm filter plate (Millipore, Sigma–Aldrich) before being loaded onto Evotip Pure trap columns.

For SILAC H4IIE phosphoproteome, reduction and alkylation were performed by the addition of 5 mM TCEP (Sigma-Aldrich) and 4.5 mM CAA diluted in 100 mM TEAB buffer. Samples were digested with Lys-C (1:100) at room temperature for 4 h and subsequently diluted in 10 mM Tris, pH 8, to a final guanidine hydrochloride concentration of 1.5 M. Next, digestion with sequencing grade modified trypsin 1:100, was performed overnight at 37ºC. Samples were acidified with TFA and centrifuged at 3000g for 10 min. The peptide mixtures were desalted and cleaned using SepPak with the final wash was done in MilliQ H2O and peptides were eluted in 50% ACN. The eluted peptides were lyophilized before phosphopeptide enrichment.

pTyr IP was performed using 120 µL Tyr1000 and 40 µL pTyr100 Ab combined per pull-down. Lyophilized samples were reconstituted in 1.4 mL of IP buffer provided with the kit and performed according to the manufacturer’s protocol. The pTyr enriched samples were purified on a homemade C8 STAGE-Tip and analyzed by LC-MS/MS. The remaining sample was high-pH (HpH) fractionated followed by titanium dioxide (TiO2) enrichment performed as previously described^68^. In short, samples were eluted from the Sep-Pak and lyophilized to a remaining volume of approximately 1 mL. The samples were fractionated using a Waters XBridge C18 3.5 μm, 4.6 × 250 mm column on an Agilent 1290 Infinity HPLC system connected to a Rheodyne MX Series II valve for sample injection. The system was operating at 1 mL/min. Buffer A was 10 mM ammonium hydroxide and buffer B was 90% ACN and 10 mM ammonium hydroxide. Samples were loaded into the column at 1 mL/min for 15 min in 1% buffer B followed by the gradient: (1–25% B in 50 min, 25–60% B in 4 min, 60–70% B in 2 min 70% B in 5 min, and 1% B for 4 min)

The HpH fractions were lyophilized and combined into 12 fractions. Phosphopeptides were enriched using metal oxide affinity enrichment (MOAC) with 5 μm Titansphere TiO2 beads (GL Sciences, Japan) as described elsewhere^69,70^. The TiO_2_ beads (3.5 mg per 1 mg sample) were incubated in 2,5-dihydroxybenzoic acid (DHB) (0.02 g/mL in 80% ACN, 6% TFA) for 20 min. The samples were lyophilized and reconstituted in 1 mL 80% ACN, 6% TFA and next incubated with freshly prepared TiO_2_ in DHB for 30 min at room temperature. The supernatant was collected for each fraction and pooled into three samples for a second round of TiO_2_ enrichment, which afterward was pooled into a single sample and subjected to a third round of TiO_2_ enrichment. The TiO_2_ beat pellets from the 16 TiO2 enriched samples were washed on a C8 STAGE-tip with 10, 40, and 80% ACN in 6% TFA, respectively. Next, the enriched phosphopeptides were eluted with 5% ammonium hydroxide, followed by 10% ammonium hydroxide with 25% ACN. The samples were lyophilized to a remaining 4 µL and 20 µL 5% ACN, 1% TFA was added to each sample, and the samples were purified on C18 STAGE-tips before the LC-MS/MS analysis.

For SILAC proteome samples were reduced using 5 mM dithiothreitol (DTT) and heating of the samples to 60 ºC for 30 min and subsequent alkylation with 10 mM CAA for 30 min at room temperature. Samples were incubated with Lys-C (1:360 w/w) at 30 ºC for 3 h followed by incubation with trypsin (1:100, w/w) at 37 ºC overnight. To remove the RapiGest, TFA was added to a final concentration of 0.5% and heated to 37 ºC for 30 min and the supernatant was collected after centrifuging for 10 min at 13,000 rpm. Samples were cleaned on Sep-Pak columns before fractionation. HILIC fractionation of the peptides was performed using a TSK-Gel amide-80 3 μm 2 × 150 mm SILICA HPLC column (Tosoh Bioscience) connected to a 1290 Infinity Binary LC system from Agilent Technologies (Santa Clara). The system was operating at 0.2 mL/min. Buffer A (0.1% formic acid (FA)) and buffer B (98% ACN with 0.1% FA). Approximately 200 µg digested protein was cleaned by SepPac, lyophilized, and reconstituted in 4 µl buffer A and 36 µl buffer B by mixing at 1100 rpm for 30 min. The samples were loaded onto an HPLC column at a flow rate of 0.2 mL/min for 5 min using 5% buffer A. The gradient progressed from 5 to 100% A for 47 min, 100-5% A for 1 min, and 5% A for 15 min. Fractions were pooled into 10 fractions, lyophilized, and reconstituted in 12 µL 0.1% FA and 1% TFA for 30 min mixing at 1200 rpm. 5 µL of each sample were then analyzed by LC-MS/MS.

### Liquid chromatography-tandem mass spectrometry and data analysis

All HepG2 IGF1R KO proteomics samples were analyzed using the Evosep One system, coupled with the Orbitrap Exploris 480 MS (Thermo Fisher Scientific) using Xcalibur tune v.1.1. A 15 cm, 150 μm inner diameter capillary column packed in-house with 1.9 μm Reprosil-Pur C18 beads (Dr. Maisch) was used. The analysis was done using pre-programmed gradients of 30 samples per day (30SPD) for the interactome and single-shot proteome, and 60 samples per day (60SPD) for phosphoproteome analysis. The column temperature was set to 60 °C using the integrated PRSO-V1 column oven (Sonation).

The spray voltage was set to 2 kV, the funnel RF level was 40, and the heated capillary temperature was 275 °C. All analyses were acquired in DIA mode, full MS resolution was set to 120,000 at *m/z* 200. The full MS AGC target was 300% with an injection time (IT) of 45 ms, and the mass range was 350–1400 *m/z*. The AGC target value for fragment spectra was 100%. In DIA analysis, 49 windows of 13.7 *m/z* were scanned from 361 to 1033 *m/z*, with a 1 Da overlap. The resolution for these scans was 15,000, with an IT of 22 ms, and the normalized collision energy was 27%. For phosphoproteome analysis using DIA, 17 windows of 39.5 *m/z* were scanned from 472 to 1143 *m/z*, with a 1 *m/z* overlap. The resolution for these scans was 45,000, with an IT of 86 ms, and the normalized collision energy was 27%. All data were acquired in positive ion and profile mode.

The acquired DIA raw data were analyzed using Spectronaut v.17.0 with a library-free approach (directDIA)^71^. The analysis utilized the SwissProt human database, UP000005640_9606 release 2018, with signal peptides removed and a separate database of common contaminants containing 20,397 and 246 sequences, respectively. The default Spectronaut Orbitrap MS mass tolerance (40 ppm) was used at MS1 and MS2 levels. The default 1% false discovery rate cutoff at precursor and protein levels was applied. For interactome and proteome analysis, fixed modifications included carbamidomethylation of cysteine, while protein N-terminal acetylation and oxidation of methionine were set as variable modifications. For the interactome searches, cross-run normalization was disabled and the protein LFQ method was changed to QUANT 2.0. For phosphoproteome analysis, phosphorylation of serine, threonine, and tyrosine were included as variable modifications.

All H4IIE SILAC samples were analyzed on an Easy-nLC 1000 connected to a Q-Exactive Orbitrap Plus (Thermo Scientific). The peptides were separated on C18-bead columns packed in-house. Analyzed with a 160 min gradient for the proteome analyses (4–25% buffer B in 120 min, 25–40% buffer B in 20 min, 40–80% buffer B in 2 min), a 240 min gradient for the pTyr samples (4–25% buffer B in 190 min, 25–40% buffer B in 30 min, 40–80% buffer B in 2 min, and a 165 min gradient for the TiO_2_ enriched phospho-samples (4–25% buffer B in 120 min, 25–40% buffer B in 25 min, 40–80% buffer B in 2 min).

The Q-Exactive mass spectrometer was operated in positive ion mode with a capillary temperature of 275 ºC. The data were acquired in data-dependent acquisition (DDA) mode with a loop count of 12. The resolution for the full scan was 70,000, AGC target of 3E6, maximum IT 20 ms, and 1 microscan. The MS/MS scans for the phospho-samples were recorded with a resolution of 35,000, AGC target of 1E6, maximum IT 120 ms, isolation window 2.2 *m/z*, NCE of 25, and underfill ratio 1.0% (intensity threshold of 8.3E4). The dynamic exclusion was set to 20 s. For the proteome samples, the MS/MS scans were recorded with a resolution of 17,500, AGC target of 5E5, maximum IT 50 ms, isolation window 2.2 *m/z*, NCE of 25, and underfill ratio 1.0% (intensity threshold of 1E5). The dynamic exclusion was set to 20 s.

Raw MS files from the H4IIE SILAC phosphoproteome and proteome analysis were processed using the MaxQuant software^72^ version 2.1.4.0. The precursor MS signal intensities were determined and the SILAC triplets were automatically quantified. Proteins were identified by searching the complete rat UniProt database, UP000002494_10116 release 2022 containing 22,825 sequences. The re-calibrated spectra were matched with a precursor mass tolerance of 4.5 ppm and 20 ppm for the fragment ions. The proteome was searched with carbamidomethyl of cysteine as a fixed modification and oxidation of methionine and acetyl protein N-term as variable modifications. The peptide and protein false discovery rate (FDR) was set at 1%. For the phospho-samples, phosphorylation of serine, threonine, and tyrosine was included as variable modifications, and PSM FDR was set at 1%. Match between runs, with a window of 0.7 min, and a maximum of 2 missed cleavages were activated for proteome and phosphoproteome data.

### Bioinformatics analysis of proteomics data

All datasets were initially filtered to remove proteins identified as contaminants from the previously mentioned supplementing common contaminant database before downstream analysis.

For the IR interactome, all files were searched in Spectronaut. Subsequently, the data was log-transformed, filtered, normalized, and imputed using the ProStaR v.1.30.7 online software tool^73^. Samples were grouped and filtered to include protein IDs identified in at least 4 out of 5 samples for at least one condition (Table S1). Normalization was performed using the global quantile alignment method. Imputation was carried out in two steps using the structured least square adaptive method for partially observed values (POVs) and DetQuantile for missing values on entire conditions (MEC) partially observed values and DetQuantile for missing values on an entire condition. The identified proteins were inputted into Workflow 1 in the online CRAPome database v.2.0 interface, using H. sapiens as the organism and Single Step Epitope tag AP-MS as the experiment type. Proteins annotated with an average score above 3 based on 718 datasets were excluded^31^. Based on DeepLoc prediction of subcellular localization, extracellular and nuclear proteins were excluded^32^.

For the dynamic interactome, data were processed in ProStaR as described above, however with insulin-sensitive and insulin-resistant samples processed individually to account for uneven efficiency. Volcano plots were generated to visualize the differential recruitment of interactors upon insulin stimulation with different concentrations. The plots were created by plotting the -log10-transformed p-values derived from a two-sided t-test against log2-transformed fold changes. Statistical significance was determined based on a hyperbolic curve threshold with s0 = 0.1 and FDR < 0.05, derived from statistical analysis using the Perseus v.1.6.5.0 software^74^ (Table S1).

For the phosphoproteomics and single-shot proteomes, cross-run normalization was performed in the Spectronaut software. For the phosphoproteomics dataset, phosphorylation sites with a localization probability score of ≥ 0.85 were log-transformed using ProStaR, followed by grouping into conditions and filtering for protein identification in minimum 3 out of 5 samples for at least one condition (Table S2). To account for the intrinsic complexity of DIA spectra and increase confidence in the site localization of phosphorylation sites, the site probability cut-off was set to 0.85. Imputation was conducted using SLSA for POVs and DetQuantile for MEC. Median subtraction was performed, based on biological experiments before the generation of volcano plots to illustrate the differential regulation of phosphorylation sites upon insulin stimulation with different concentrations for insulin-sensitive and insulin-resistant samples and between insulin-sensitive and -resistant cells directly. Statistical significance was defined such that phosphorylation sites were found to be significantly regulated when the change was minimum 2-fold and p-value < 0.05, or as specified in the respective Fig. legends.

The single-shot proteome was log2-transformed using the Perseus software, and median subtraction was performed, based on biological experiment. Volcano plots were generated as described above. The 5-minute insulin stimulation was not considered in the analysis of differentially regulated proteins. For the co-IP lysis buffer proteome, the cutoffs for both directions were set to a ratio cutoff of 1.25 and FDR < 0.05. For the SDS-based proteome, statistical significance was determined based on a hyperbolic curve threshold with s0 = 0.1 and FDR < 0.05 derived from statistical analysis using Perseus (Table S3). The protein association network based on IR interactome data was obtained using the STRING database v.11.5^75^. Default search parameters were used in the multiple protein tab. The network was visualized using Cytoscape v.3.10.0^76^. The KEGG pathway enrichment analyses were performed using the InnateDB resource v.5.4^77^. The pathway overrepresentation analysis was conducted using the hypergeometric algorithm, with Benjamini-Hochberg FDR correction (Ben. Ho. FDR corrected). Enriched pathways were defined with pathway ORA p-value < 0.05. If multiple enriched pathways had an identical profile of identified involved proteins, only the entry with the lowest pathway ORA p-value was included.

The H4IIE cell SILAC proteome and phosphoproteome datasets were filtered for contaminants and reverse hits. For the proteome analysis with 24- and 48-hour insulin and S597, treatment ratios and intensities were log2-transformed. Normalized ratios and intensities were reported as the median of three replicates. Proteins identified by only one peptide were excluded, and data were further filtered to include proteins with at least two valid values (of the three replicates) in at least one of the treatment groups. Statistically significant changes in protein ratio induced by long-term treatment with insulin or S597 were determined using significance B testing (*p* < 0.05) with the Perseus software (Table S4).

For the DDA SILAC phosphoproteomics dataset, only phosphorylation sites defined based on a phosphorylation site localization probability ≥ 0.75 (class I, as defined by Olsen et al.^78^). were included. Normalized SILAC phosphorylation site ratios and intensities were reported as the median of two replicates, and the data was log2-transformed. The table was expanded to include singly, doubly, and triply phosphorylated sites. Only relative changes compared to untreated controls were used; therefore, H/M SILAC ratios were not included in the downstream analysis. A cutoff of > 2-fold regulation, if valid in at least one of the treatment groups, was applied as section criteria (Table S4).

## Electronic Supplementary Material

Below is the link to the electronic supplementary material.

Supplementary Material 1

Supplementary Material 2

Supplementary Material 3

Supplementary Material 4

Supplementary Material 5

## Data Availability

The DIA and DDA MS proteomics data have been deposited to the ProteomeXchange Consortium via the PRoteomics IDEntifications (PRIDE)^79^ partner repository with the dataset identifier PXD047011 and PXD046865, respectively.
