# Supplementary material for: Multi-layered proteomics identifies insulin-induced upregulation of the EphA2 receptor via the ERK pathway which is dependent on low IGF1R level
Source: Sci Rep. 2024 Nov 21;14:28856. doi: 10.1038/s41598-024-77817-5 (PMC11582730; doi:10.1038/s41598-024-77817-5)
Supplement: Supplementary file 5 — Supplementary Material 5 [file 41598_2024_77817_MOESM5_ESM.pdf]

Supplementary information for

Multi-layered proteomics identifies insulin-induced upregulation of the EphA2 receptor via the ERK pathway which is dependent on low IGF1R level

Sarah Hyllekvist Jørgensen[1,2], Kristina Bennet Emdal[1,*], Anna-Kathrine Pedersen[1], Lene Nygaard Axelsen[3], Helene Faustrup Kildegaard[4], Damien Demozay[5], Thomas Åskov Pedersen[5], Mads Grønborg[3], Rita Slaaby[5], Peter Kresten Nielsen[2], Jesper Velgaard Olsen[1,*]

*For correspondence: kristina.emdal@cpr.ku.dk; jesper.olsen@cpr.ku.dk

[1] Proteomics Program, Novo Nordisk Foundation Center for Protein Research, Faculty of Health and Medical Sciences, University of Copenhagen, Copenhagen DK-2200, Denmark
[2] Novo Nordisk A/S, Global Research Technologies, Maaloev DK-2760, Denmark
[3] Novo Nordisk A/S, Global Translation, Maaloev DK-2760, Denmark
[4] Novo Nordisk A/S, Global Nucleic Acid Therapies, Maaloev DK-2760, Denmark
[5] Novo Nordisk A/S, Global Drug Discovery, Maaloev DK-2760, Denmark

Supplementary figure 1

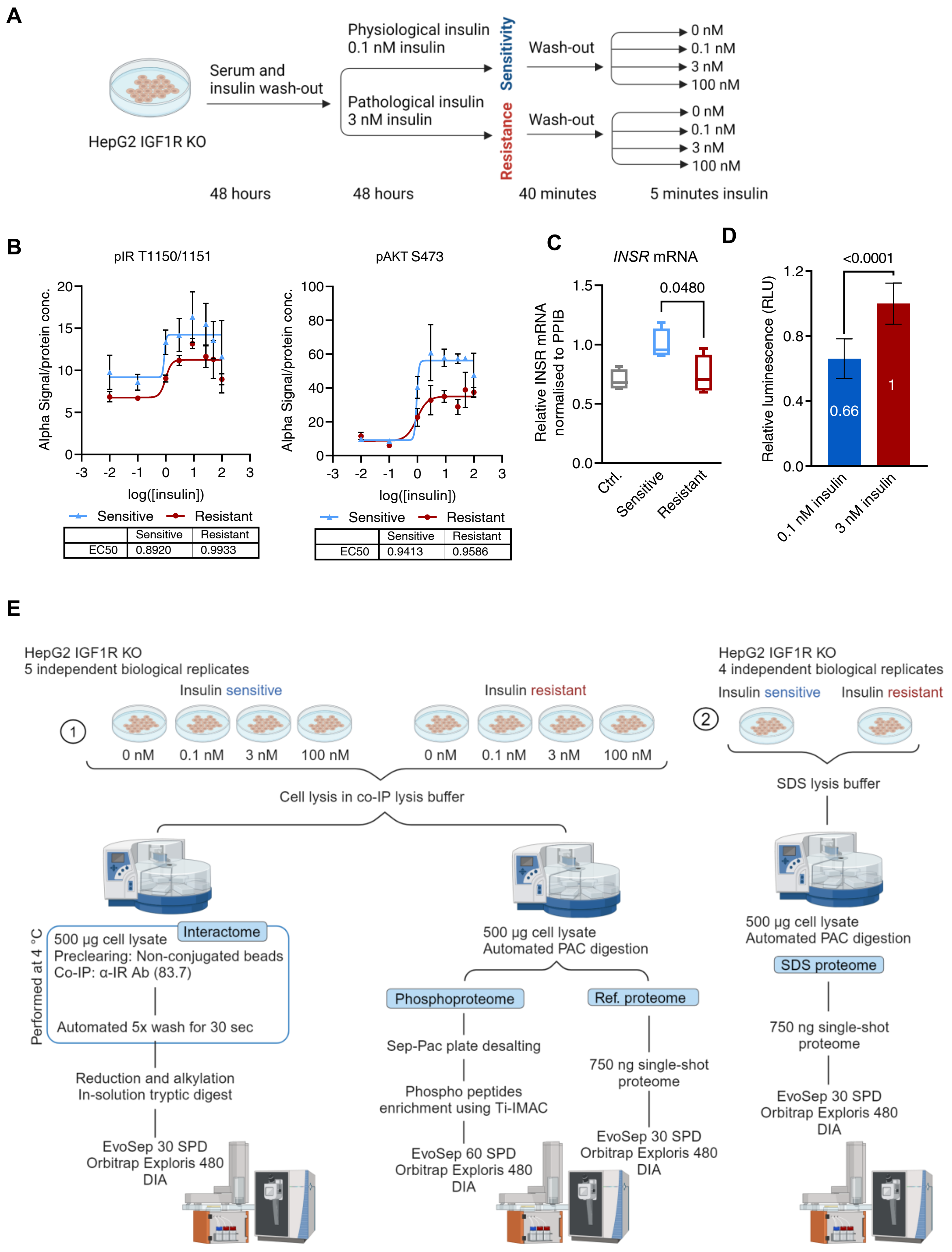

Figure S1. Validation of insulin induced insulin resistance in HepG2 IGF1R KO cell model.

A. Schematic of the insulin resistance inducing protocol in the HepG2 IGF1R KO cell model, adapted from Dall'Agnese et al. (ref.) B. Insulin dose-response curves for insulin-sensitive and resistant HepG2 IGF1R KO cells after stimulation with 0-600 nM insulin. The calculated EC50-values are indicated below the curves. Representative example of n=3 independent biological experiments with 3 technical replicates. C. qPCR of FASN mRNA levels in HepG2 IGF1R KO cells the insulin-resistance inducing cell treatment of HepG2 IGF1R KO cells. Control (ctrl.) 48-hour serum starvation. Mean of n=4 biological independent experiments with n=6 technical replicates relative to the insulin sensitive condition (two-sample unpaired t test). Only significant p-values are shown. D. Relative cell viability measured in cells cultured for 48 hours with either 0.1 nM (physiological) or 3 nM insulin (pathophysiological) after 24-hour serum starvation. Cell viability was determined using the CellTiter-Glo® assay. The relative means (annotated in the respective bars) ±SD of n=3 technical replicates for n=3 biological independent experiments (two-sample unpaired t test). E. Schematic of the detailed workflow of DIA-MS-based analysis for the interactome, phosphoproteome, and reference single-shot proteome in the insulin-resistant HepG2 IGF1R KO cell model (n=5 independent biological replicates). Workflow for deep-proteome analysis (n=4 independent biological replicates) in (2).

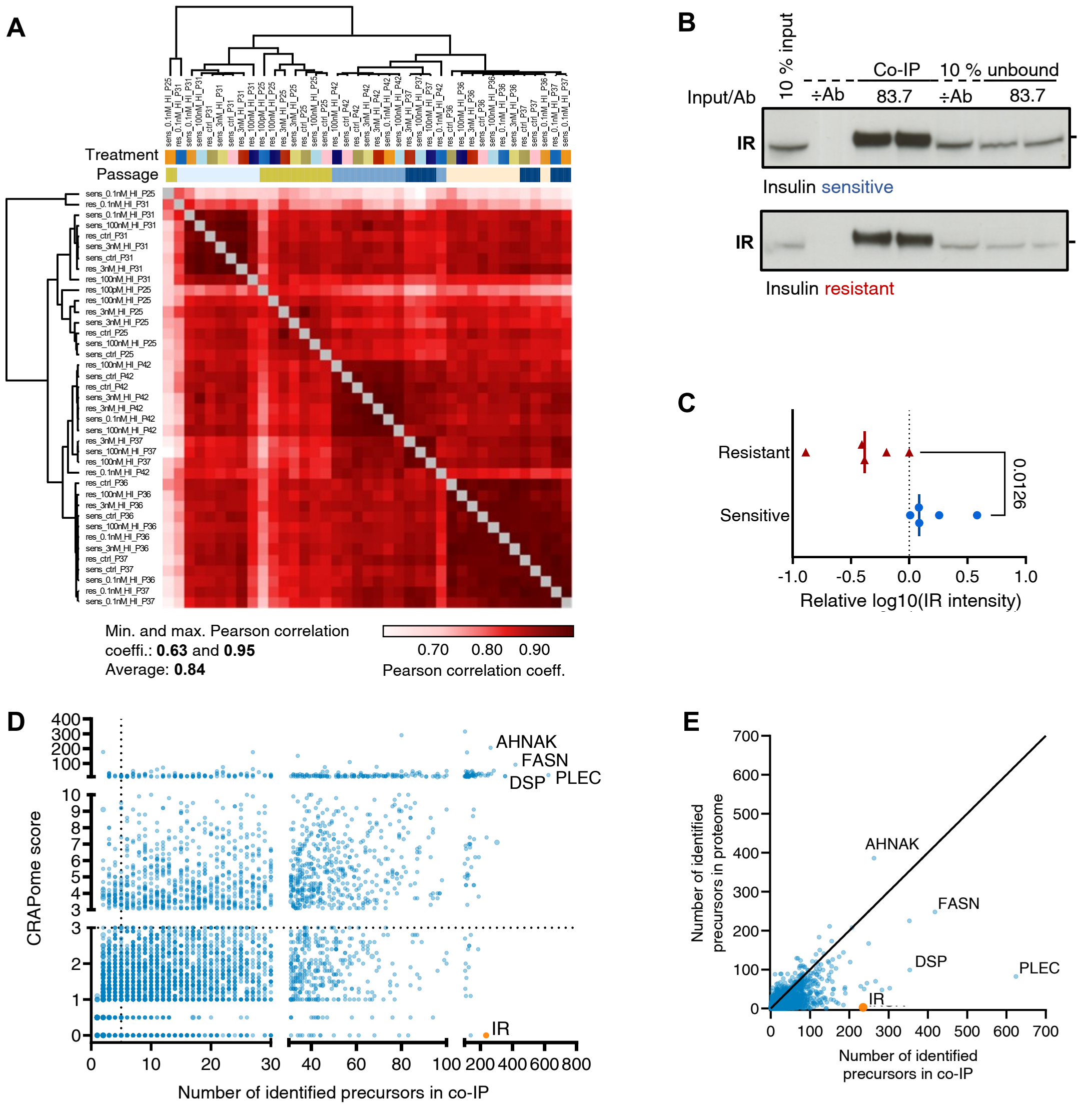
A
Treatment
Passage
Min. and max. Pearson correlation coeffi.: 0.63 and 0.95
Average: 0.84
0.70 0.80 0.90
Pearson correlation coeff.
B
10 % input
Co-IP
10 % unbound
Input/Ab
÷Ab
83.7
÷Ab
83.7
IR
97
Insulin sensitive
IR
97
Insulin resistant
C
Resistant
Sensitive
0.0126
-1.0 -0.5 0.0 0.5 1.0
Relative log10(IR intensity)
D
CRAPome score
AHNAK
FASN
DSP
PLEC
IR
Number of identified precursors in co-IP
E
Number of identified precursors in proteome
AHNAK
FASN
DSP
PLEC
IR
Number of identified precursors in co-IP

Figure S2. Quality control of interactome data

A. Heatmap showing Pearson correlation coefficients of the IR co-IP DIA-MS datasets from the insulin-resistant HepG2 IGF1R KO cell model (n=5 biological independent replicates for each condition). The samples are ordered by hierarchical clustering using the Pearson distance metric algorithm. Minimum, maximum, and average Pearson correlation coefficients are indicated. B. Immunoblot analysis in IR co-IP insulin-sensitive and -resistant HepG2 IGF1R KO cell lysates. Control without antibody (÷Ab) for co-IP was included. C. -log10-transformed MS-intensities of IR from the co-IP dataset (after median subtraction on cell passage) in insulin-sensitive and -resistant cells without insulin stimulation (two-sample unpaired t test). D. Scatter plot depicting the number of precursors against Contaminant Repository for Affinity Purification (CRAPome) scores (ref.). Identifications with low CRAPome scores and high peptide counts are less likely to be false positive IR interactors. E. Scatter plot illustrating the number of precursors identified per protein in the reference proteome against the IRinteractome datasets.

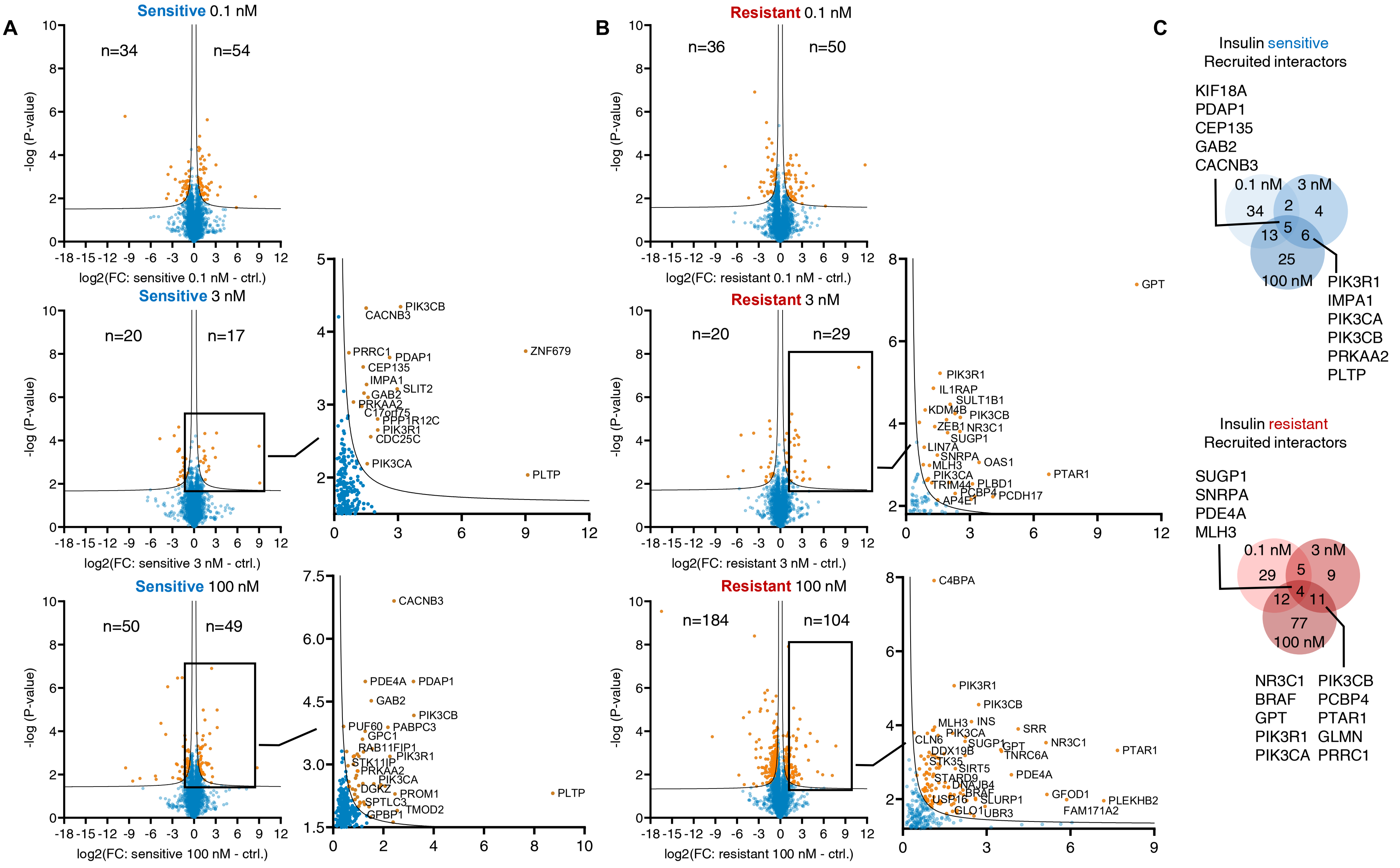
A
Sensitive 0.1 nM
n=34
n=54
-log (P-value)
log2(FC: sensitive 0.1 nM - ctrl.)
Sensitive 3 nM
n=20
n=17
log2(FC: sensitive 3 nM - ctrl.)
PIK3CB
CACNB3
PRRC1
PDAP1
CEP135
IMPA1
SLIT2
GAB2
PRKAA2
C17orf75
PPP1R12C
PIK3R1
CDC25C
PIK3CA
ZNF679
PLTP
Sensitive 100 nM
n=50
n=49
log2(FC: sensitive 100 nM - ctrl.)
CACNB3
PDE4A
PDAP1
GAB2
PIK3CB
PUF60
PABPC3
GPC1
RAB11FIP1
PIK3R1
STK11IP
PRKAA2
PIK3CA
DGKZ
PROM1
SPTLC3
GPBP1
TMOD2
PLTP
B
Resistant 0.1 nM
n=36
n=50
log2(FC: resistant 0.1 nM - ctrl.)
Resistant 3 nM
n=20
n=29
log2(FC: resistant 3 nM - ctrl.)
GPT
PIK3R1
IL1RAP
SULT1B1
KDM4B
PIK3CB
ZEB1
NR3C1
SUGP1
LIN7A
SNRPA
MLH3
OAS1
PIK3CA
TRIM44
PLBD1
PCBP4
PCDH17
AP4E1
PTAR1
Resistant 100 nM
n=184
n=104
log2(FC: resistant 100 nM - ctrl.)
C4BPA
PIK3R1
PIK3CB
MLH3
INS
SRR
PIK3CA
CLN6
SUGP1
GPT
NR3C1
DDX19B
TNRC6A
PTAR1
STK35
SIRT5
STARD9
PDE4A
DNAJB4
BRAF
GFOD1
USP16
SLURP1
PLEKHB2
GLO1
UBR3
FAM171A2
C
Insulin sensitive
Recruited interactors
KIF18A
PDAP1
CEP135
GAB2
CACNB3
0.1 nM
3 nM
100 nM
PIK3R1
IMPA1
PIK3CA
PIK3CB
PRKAA2
PLTP
Insulin resistant
Recruited interactors
SUGP1
SNRPA
PDE4A
MLH3
NR3C1
BRAF
GPT
PIK3R1
PIK3CA
PIK3CB
PCBP4
PTAR1
GLMN
PRRC1

Figure S3. IR interactome response to insulin dose treatment in insulin-sensitive and resistant HepG2 IGF1R KO cells.

A. Volcano -log10(p-value) versus log2(fold change) of protein LFQ intensity measured by MS. Illustrating the differentially recruited IR interactors, with fold-change difference between unstimulated (ctrl.) and 5-minute 0.1, 3, or 100 nM insulin stimulation, respectively, in the insulin sensitive cells (two-sided t-test in Perseus software, FDR<0.05, s0=0.1). Zoom on insulin-dependent interactors upon 3 and 100 nM insulin stimulation. B. Same as in A. under insulin resistant conditions. C. Overlap of recruited interactors after stimulation with 0.1, 3, and 100 nM insulin. Insulin sensitive (blue) and resistant (red) cells.

Supplementary figure 4

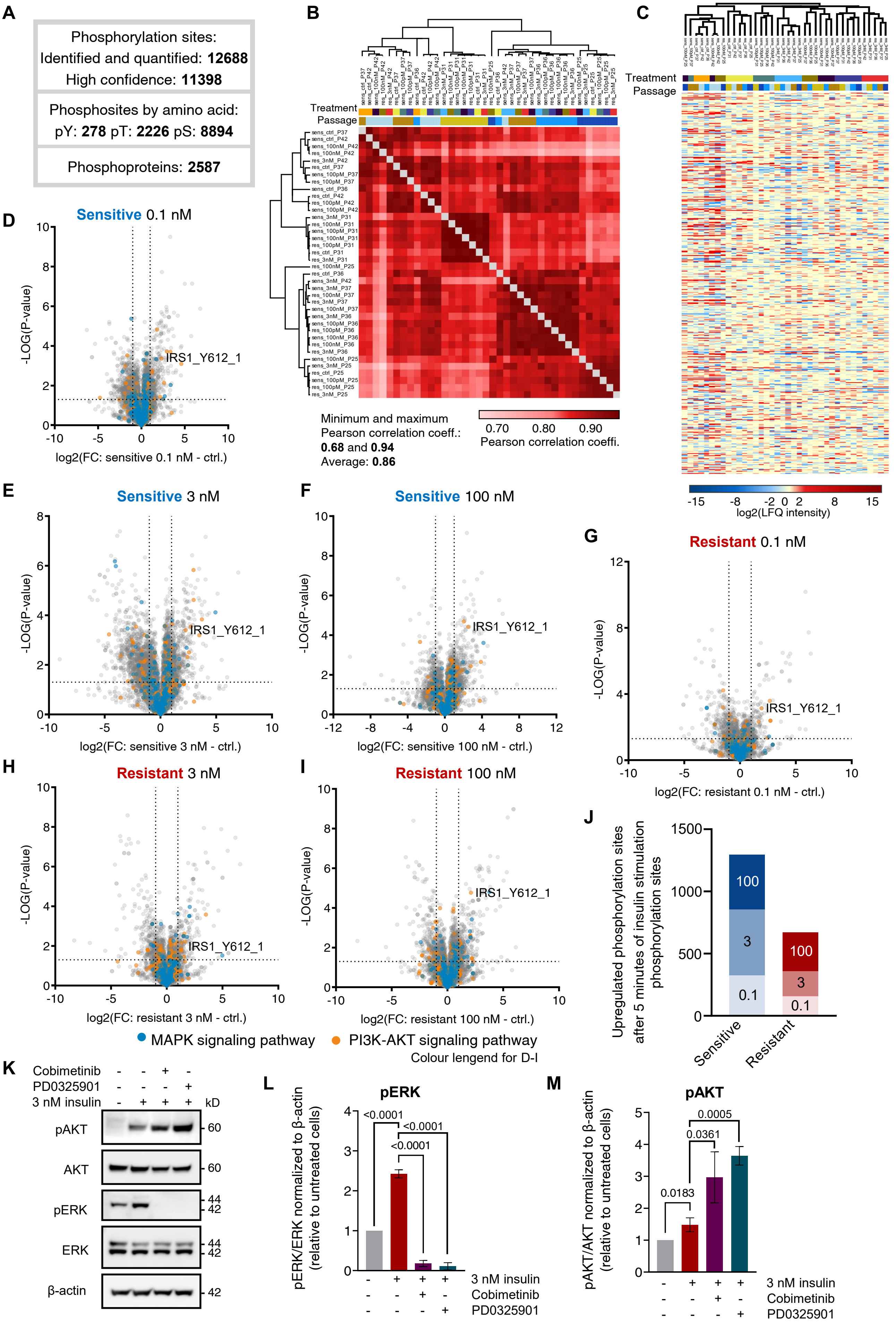

Figure S4. Data quality check of phosphoproteomics data and analysis of differential phosphoproteome response in insulin-sensitive and -resistant HepG2 IGF1R KO cells.

A. Phosphoproteome summary: Number of identified phosphorylation sites and phosphoproteins. B. Heatmap comparing the Pearson correlation coefficients of the data-independent acquisition mass spectrometry phosphoproteomics data in insulin-sensitive and insulin-resistant HepG2 IGF1R KO cells. The samples were ordered by hierarchical clustering using the Pearson distance metric algorithm. The heatmap displays the minimum, maximum, and average Pearson correlation coefficients (n=5 biological independent experiments). C. Hierarchical clustering of the log2-transformed LFQ intensities of phosphopeptides after median subtraction based on cell passages. D-I. Volcano plots showing the -log10(p-value) versus log2(fold change) of phosphorylation site intensities. The fold-change represents insulin stimulation with 0.1, 3, or 100 nM insulin for 5 minutes compared to unstimulated control in insulin-sensitive and insulin-resistant cells (p-value<0.05, 2-fold-change). Phosphosites from proteins in MAPK (blue) and PI3K-AKT (orange) signaling pathways highlighted, and specific IRS1 site is annotated in all plots. J. Column chart of number of upregulated phosphorylation sites in the phosphoproteome after insulin stimulation in insulin-sensitive and insulin-resistant cells. Bar colored based on insulin concentration used for short stimulation. K. Immunoblot of HepG2 IGF1R KO cells stimulated for 24 hours with 3 nM insulin without or with inhibition of MEK with either 1 µM cobimetinib or 1 µM PD0325901. Representative blot of n=3 independent biological replicates. L.-M. Quantification pERK and pAKT levels from immunoblot, with p-values<0.05 annotated (two-sample unpaired t-test).

A

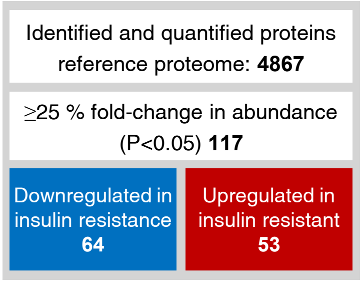

C

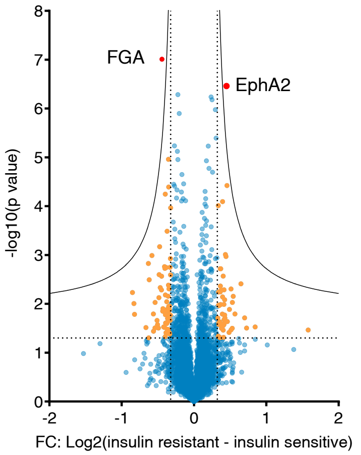

B

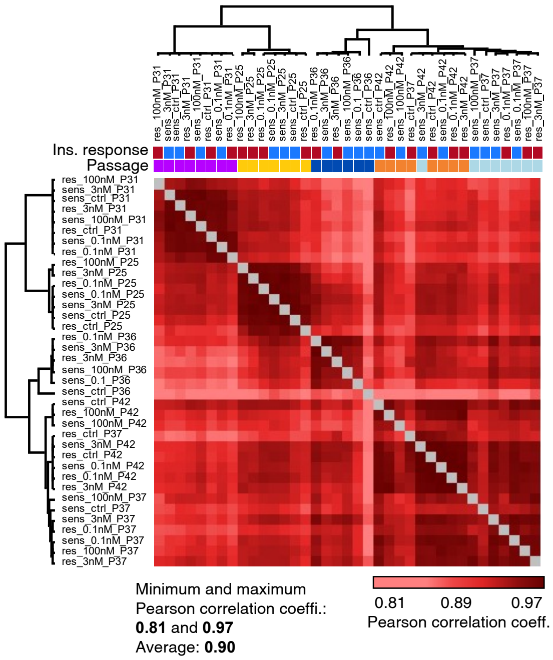

D

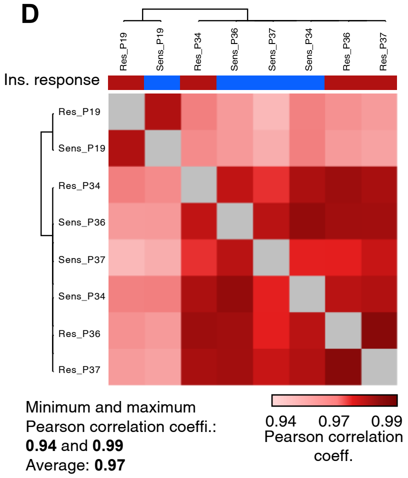

E

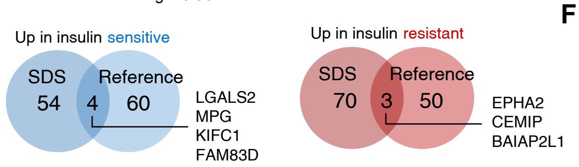

F

Gene name	No. ID peptides (Ref./SDS)	Ref. proteome (fold-change)	SDS proteome (fold-change)
LGALS2	5/10	1.50	1.47
MPG	7/9	1.25	1.22
KIFC1	5/9	1.28	1.50
FAM83D	5/3	1.29	1.32
EPHA2	27/35	1.37	1.46
CEMIP	11/27	1.36	1.35
BAIAP2L1	12/13	1.26	1.33

G

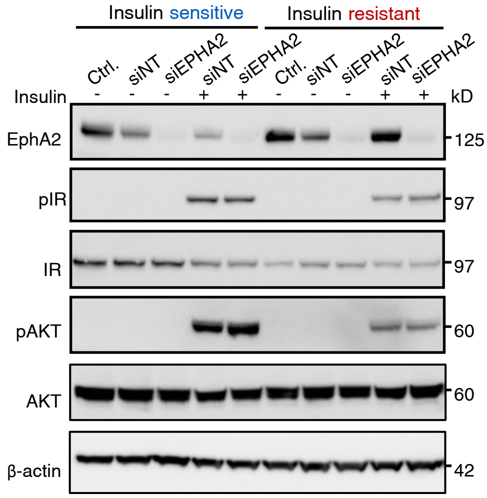

H

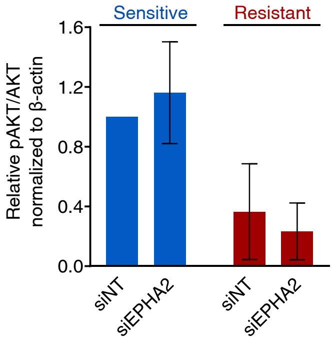

Figure S5. Data quality and comparison of reference and SDS proteome datasets with EphA2 siRNA KD follow-up.

A. Information on the reference proteome data, showing the number of identified proteins and the number of regulated proteins in insulin-sensitive and -resistant cells (≥1.25-fold change and significance, $p<0.05$). B. Volcano plot presenting differentially regulated proteins identified in insulin-sensitive and resistant reference proteome from the single-shot proteome MS analysis. Significantly regulated proteins (≥1.25-fold change and significance, $p<0.05$ dotted line) are highlighted in orange and (FDR<0.05, S0 = 0.1 solid line) in red with label (n=37). C. Heatmap comparing the Pearson correlation coefficients of the data-independent acquisition mass spectrometry single-shot proteome in insulin-sensitive and insulin-resistant HepG2 IGF1R KO. The samples were ordered by hierarchical clustering using the Pearson distance metric algorithm with the minimum, maximum, and average Pearson correlation coefficients being stated (n=5 biological independent replicates). D. Same as in C, for the SDS proteome samples. E. Overlap of proteins identified as being significantly up- or down-regulated between insulin sensitive and resistant cells in the reference and SDS proteome analysis. F. Table of the proteins identified as significantly regulated in both proteome analyses, as shown in E. It includes the number of peptides identified in the two proteomes and their respective levels of fold-change regulation in the two analyses. G. Representative immunoblot of insulin-sensitive and resistant HepG2 IGF1R KO cell lysates with EphA2 siRNA knock-down (NT=non-targeting control). Cells stimulated with 3 nM insulin are indicated (n=4 biological independent replicates) (two-sample unpaired t test). H. Quantification of phospho-AKT levels from immunoblot, shown in F, in insulin-sensitive and resistant lysates with and without EphA2 knockdown. Relative to siNT in the insulin-sensitive cells.

Supplementary figure 6

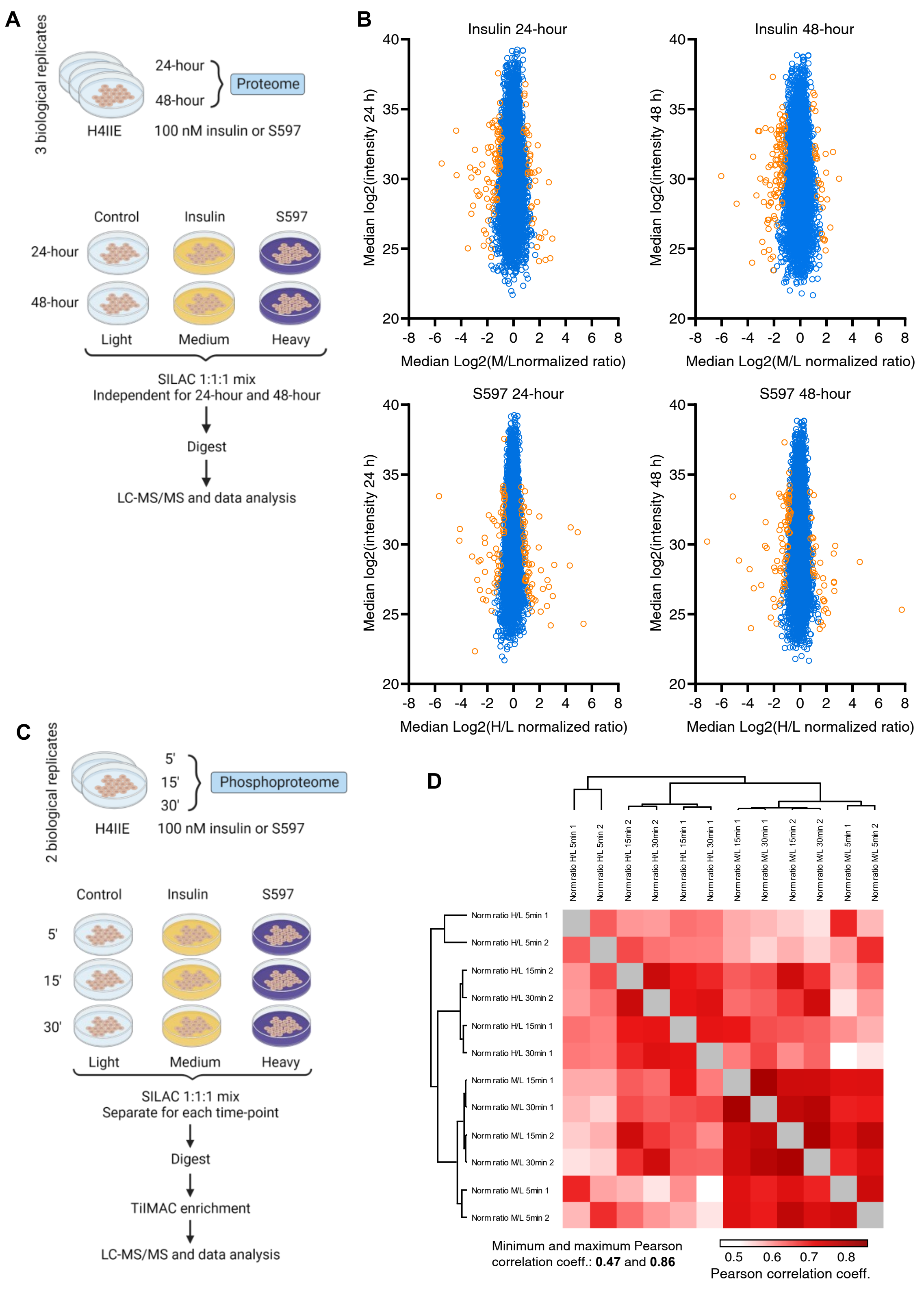

Figure S6. Experimental workflow and data quality control of SILAC MS-data from H4IIE cells.

A. Experimental design and procedure for SILAC MS-based quantitative proteomics experiment in H4IIE cells treated for 24- or 48-hours with either 100 nM insulin or the partial insulin agonist S597 (n=3 independent biological replicates). B. Plots of log2-transformed summed peptide intensities, as a function of normalized log2-transformed protein ratio for control and insulin or S597 treated cells. Proteins are colored based on p-values for regulation ($p<0.05$ in orange, and $p\geq0.05$ in blue, from significance B test, with Ben. Ho FDR<0.05 in Perseus). C. Experimental design and procedure for generation of SILAC MS-based quantitative phosphoproteomics experiment in H4IIE cells stimulated for 5-, 15-, or 30-minutes with either 100 nM insulin or S597 (n=2 independent biological replicates). D. Heatmap comparing the Pearson correlation coefficients of the SILAC phosphopeptide ratios in H4IIE after temporal stimulation with 100 nM insulin or S597. The samples were ordered by hierarchical clustering using the Pearson distance metric algorithm. The heatmap displays the minimum, maximum, and average Pearson correlation coefficients.

Fig. 1

Full length images of immunoblot data

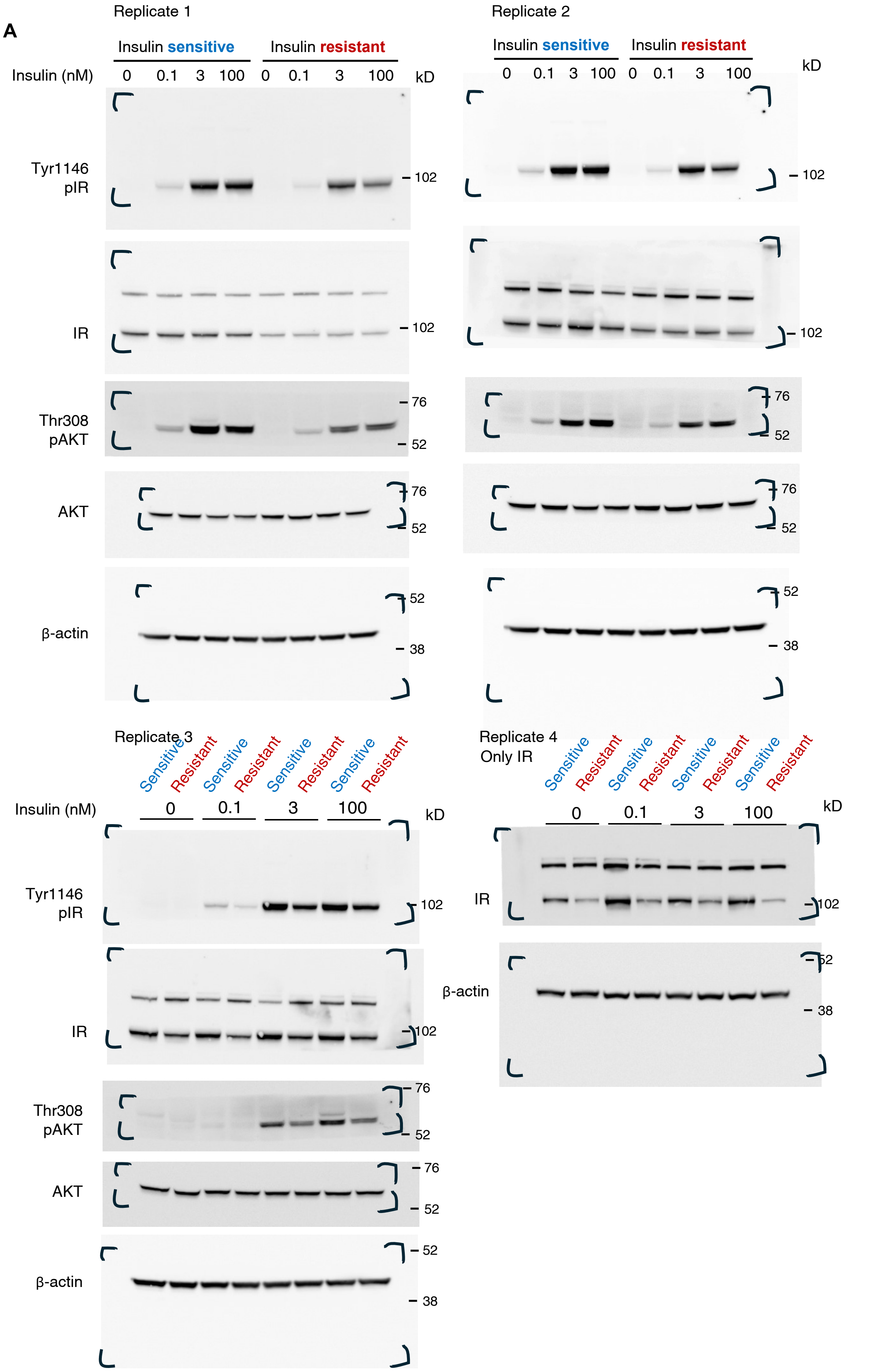

Fig. 3. EphA2 blot shown in Fig. 6.
Two biological replicates blotted on same immunoblot

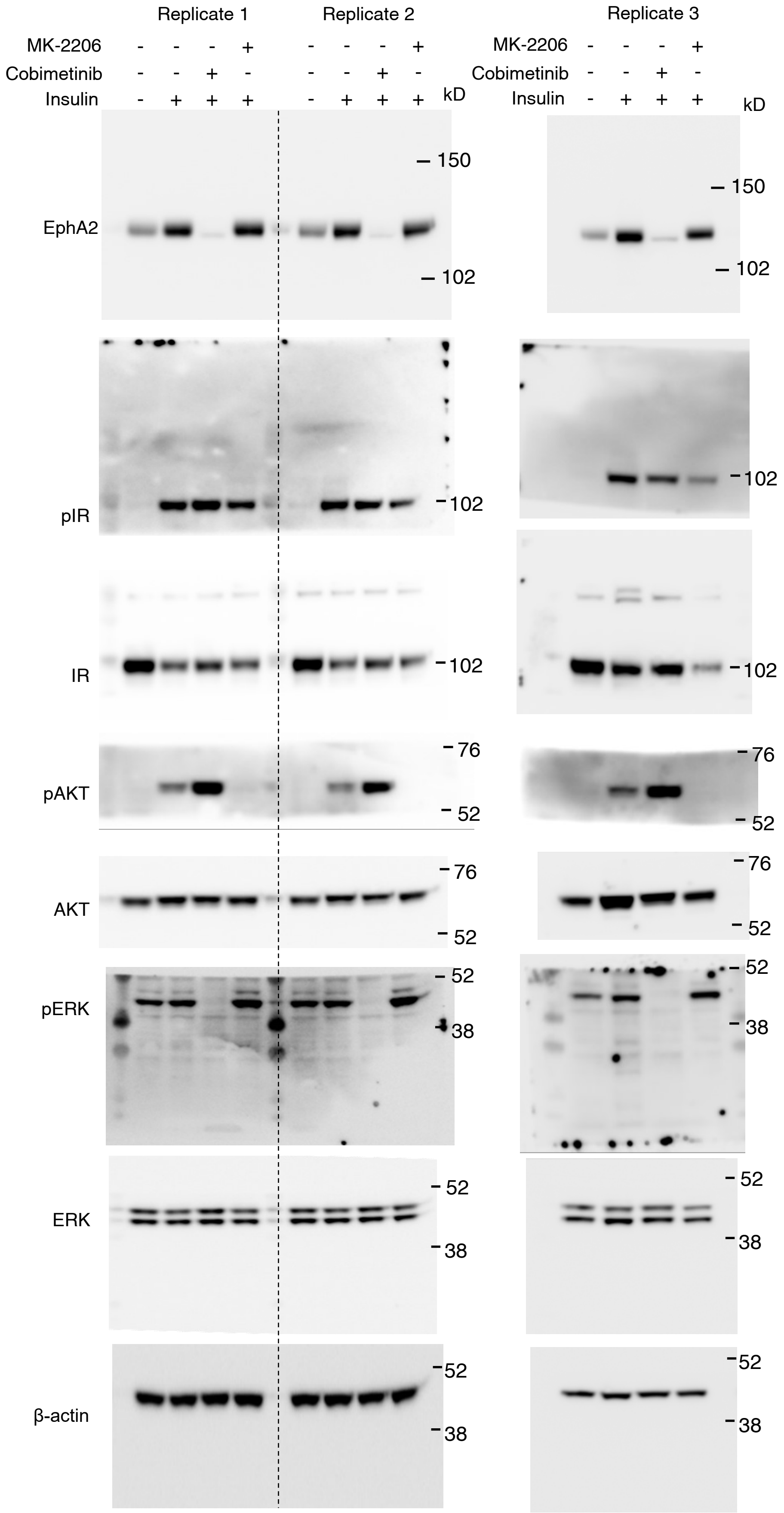

E. HepG2 IGF1R KO
Four biological replicates blotted on same immunoblot

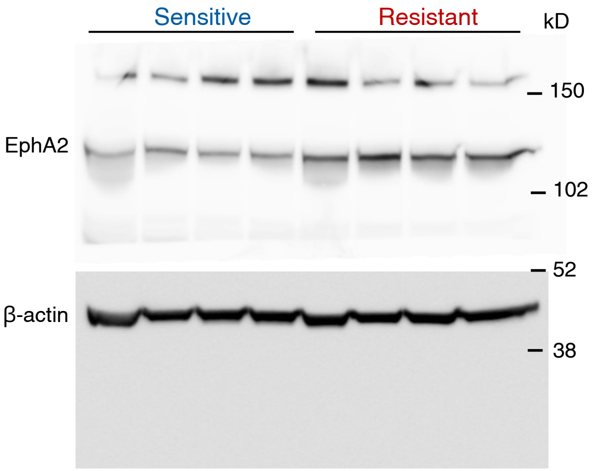

F. HepG2 IGF1R KO
Four biological replicates blotted on same immunoblot

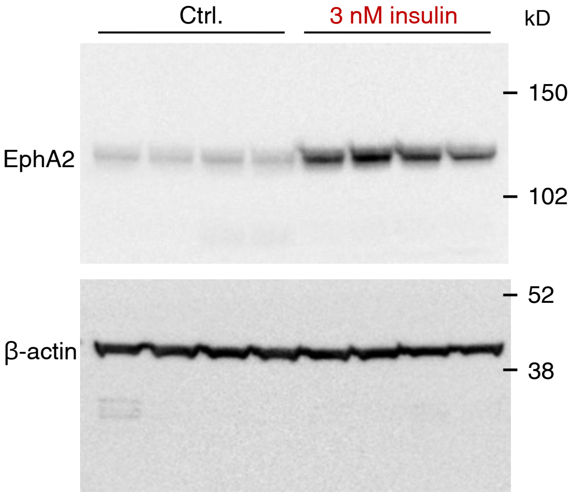

Fig. 5

Full length images of immunoblot data

A Different cell lines.
Replicate 1

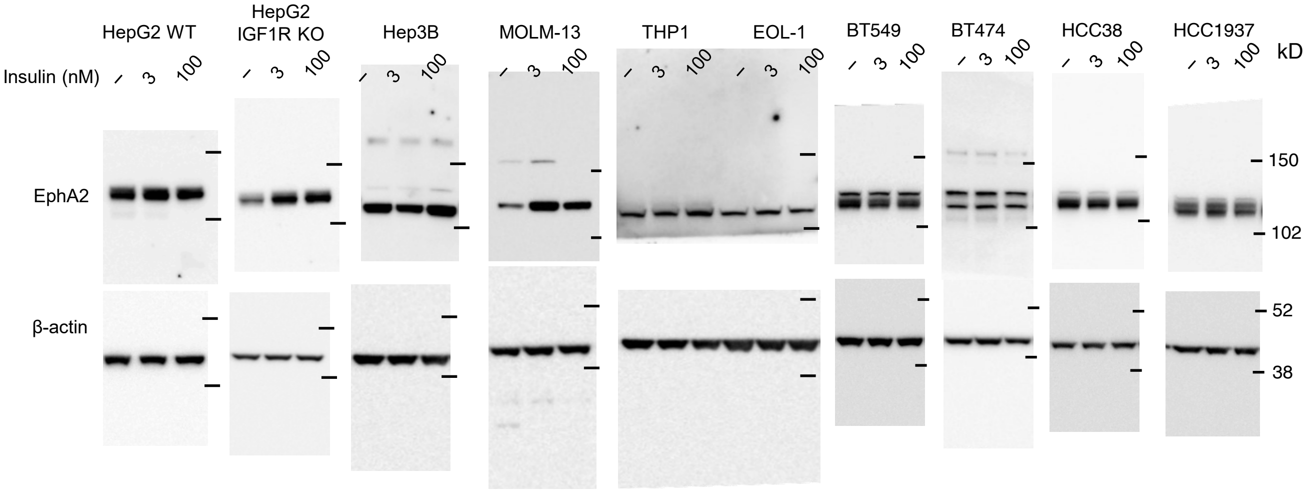

Replicate 2

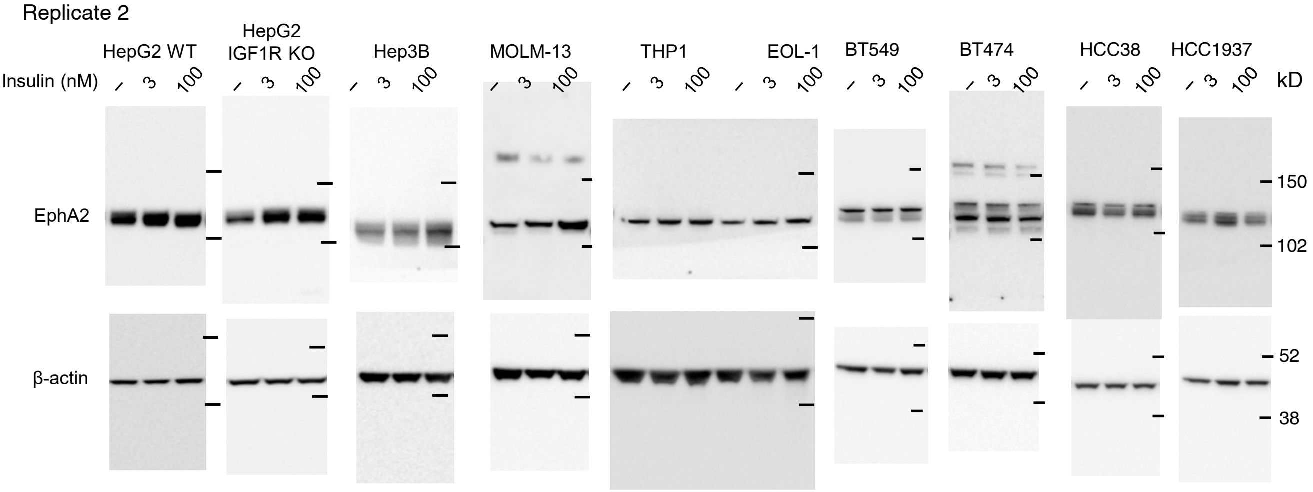

Replicate 3.
Only Hep3B and MOLM13

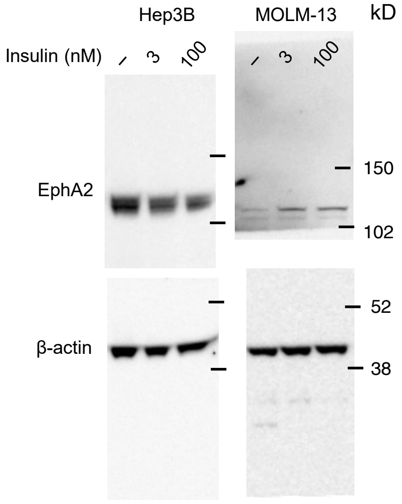

H4IIE: Three biological replicates blotted on same immunoblot
Same as in Fig 5.G

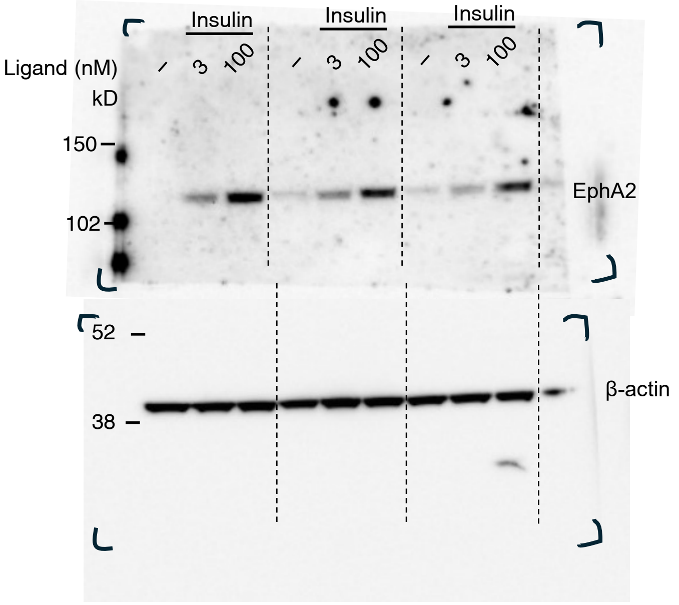

c.

Different cell lines. Replicate 1

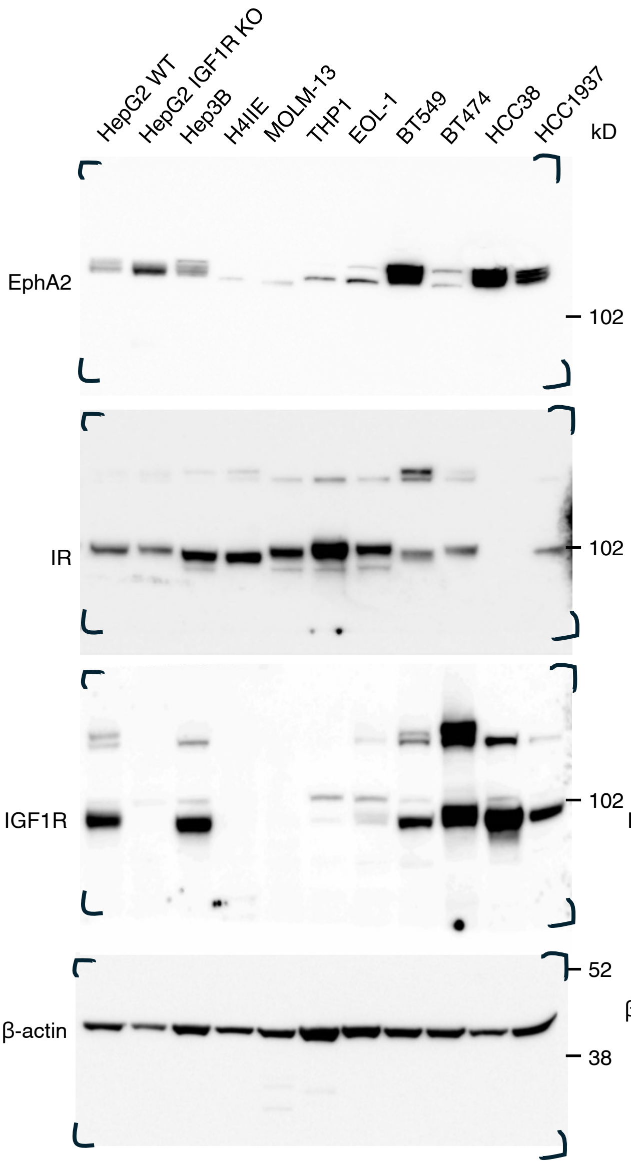

Replicate 2

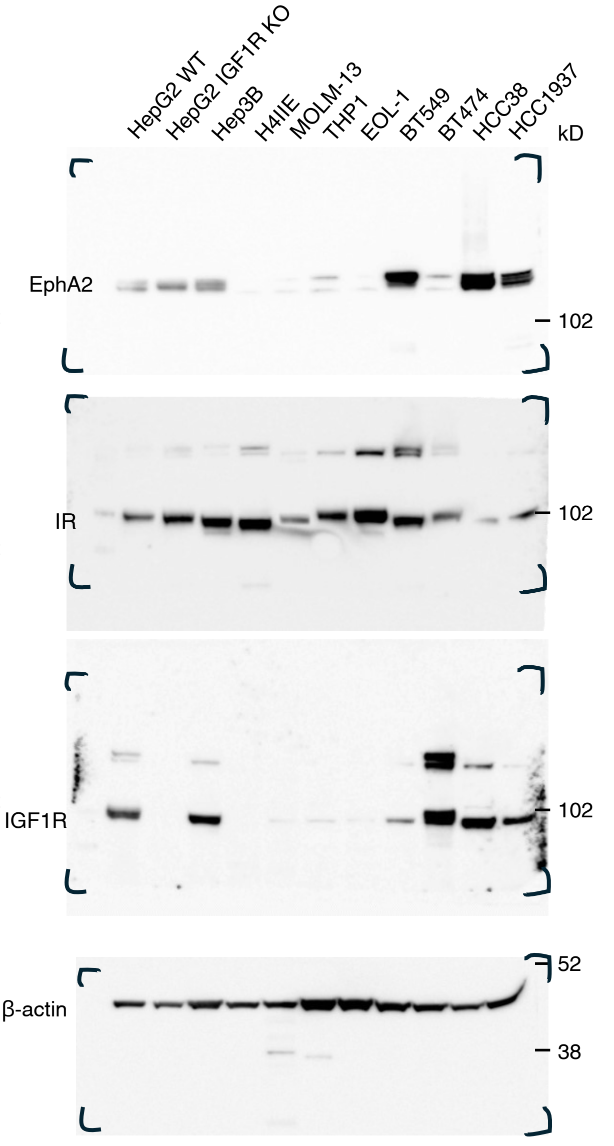

E.

HepG2 IGF1R KO.

Three biological replicates blotted on same immunoblot

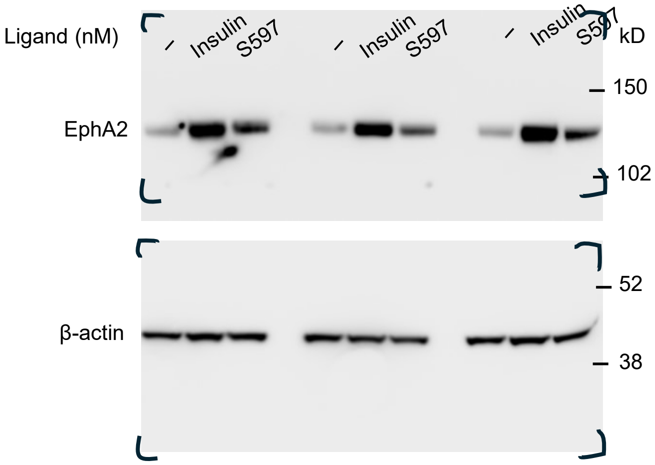

G.

H4IIE.

Three biological replicates blotted on same immunoblot

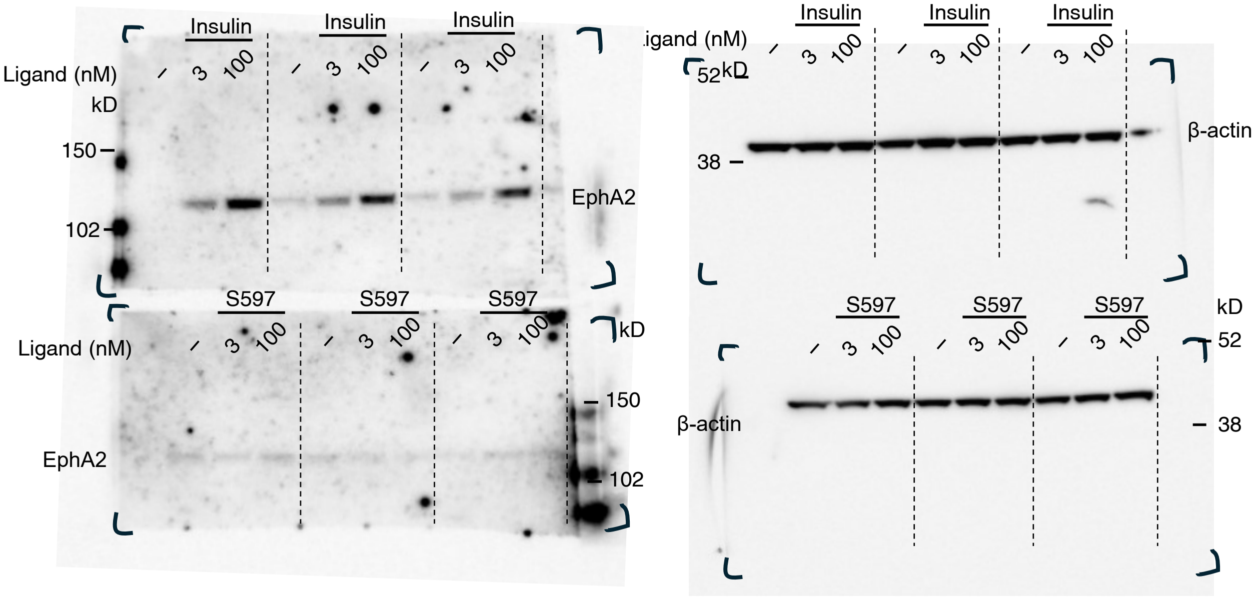

Full length images of immunoblot data

Fig. S2

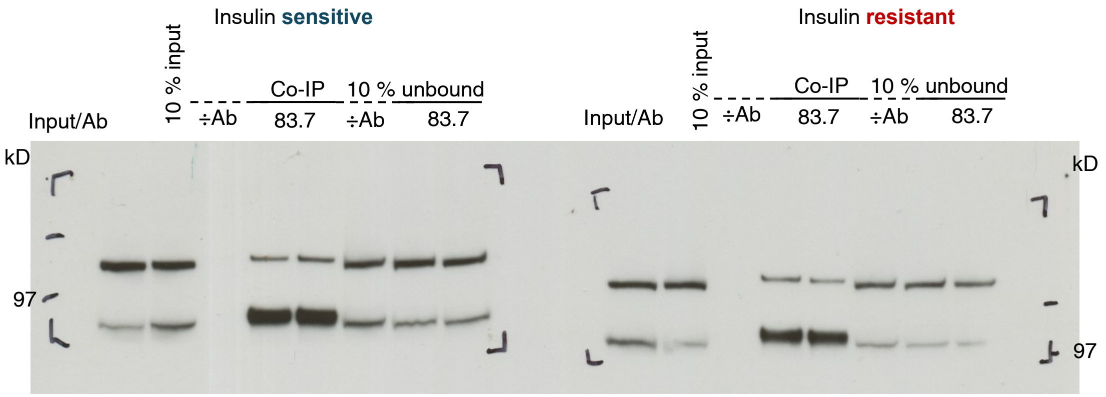

This western blot was visualized using the Novex ECL Chemiluminescent Substrate Reagent Kit (Invitrogen), and bands were detected through exposure to Hyperfilm (Amersham, GE Healthcare).

Fig. S4

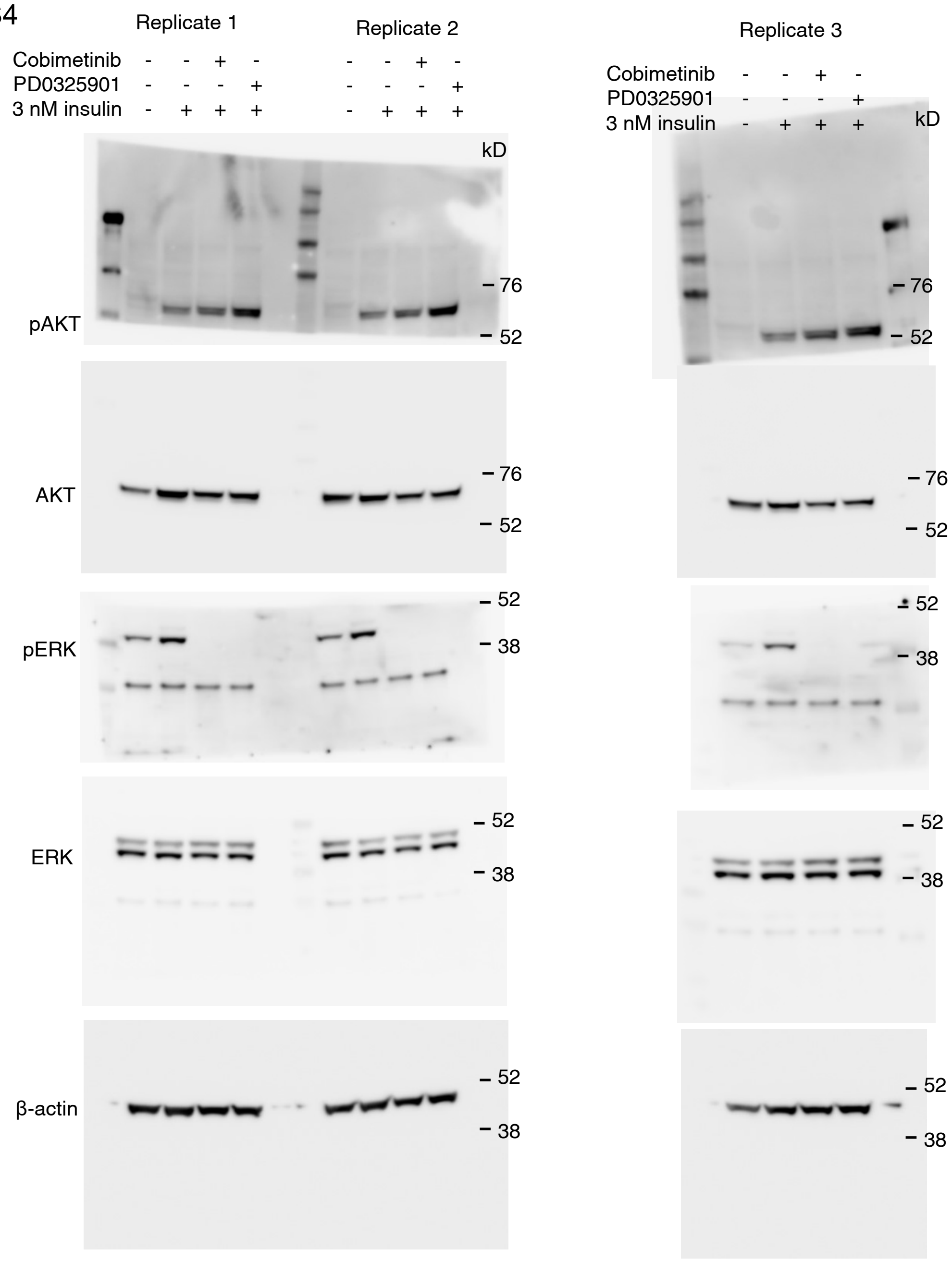

Fig. S5

Full length images of immunoblot data

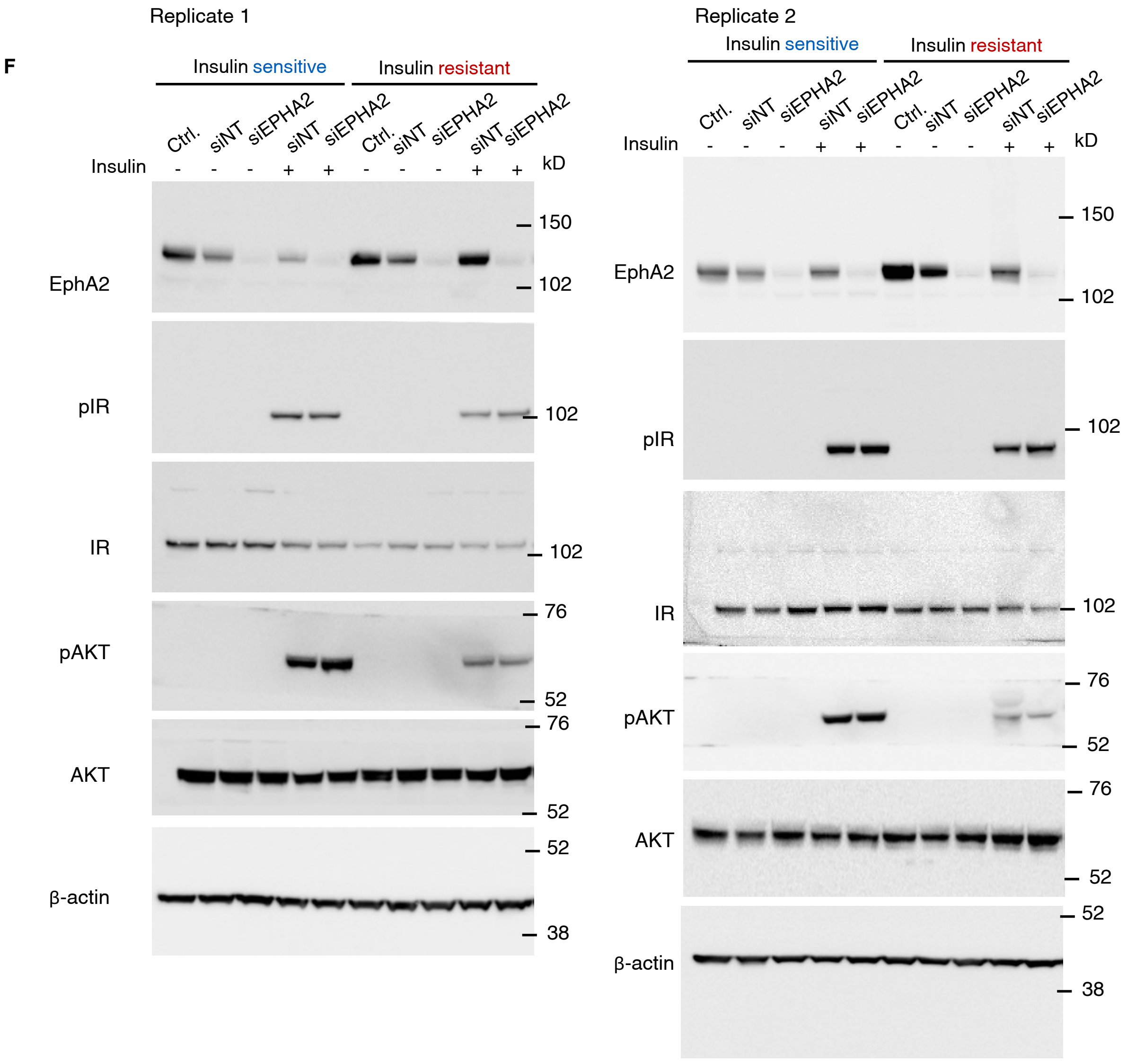

Fig. S5

Full length images of immunoblot data

F

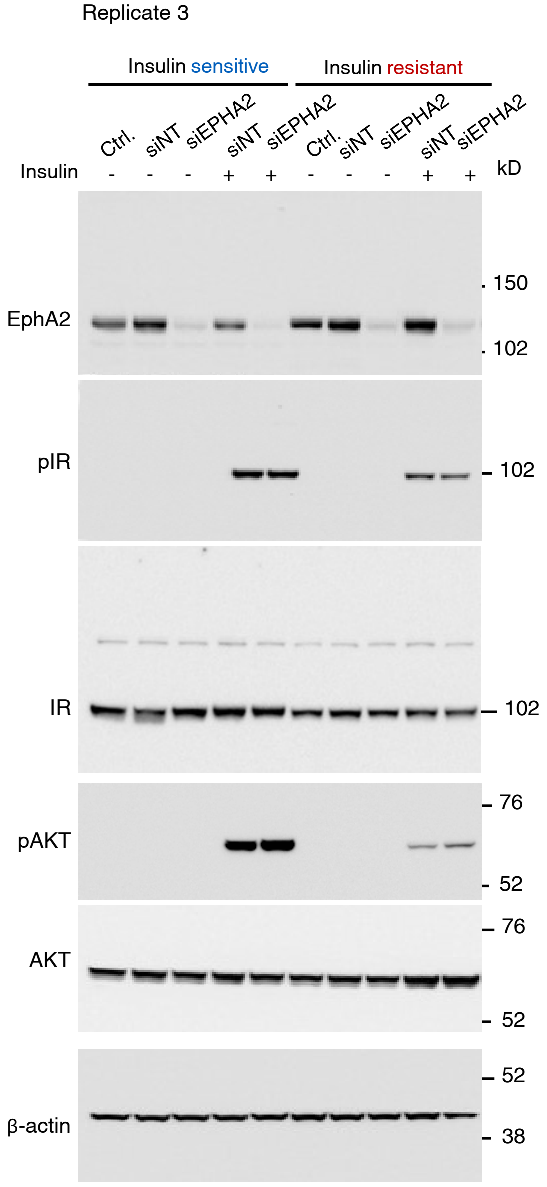

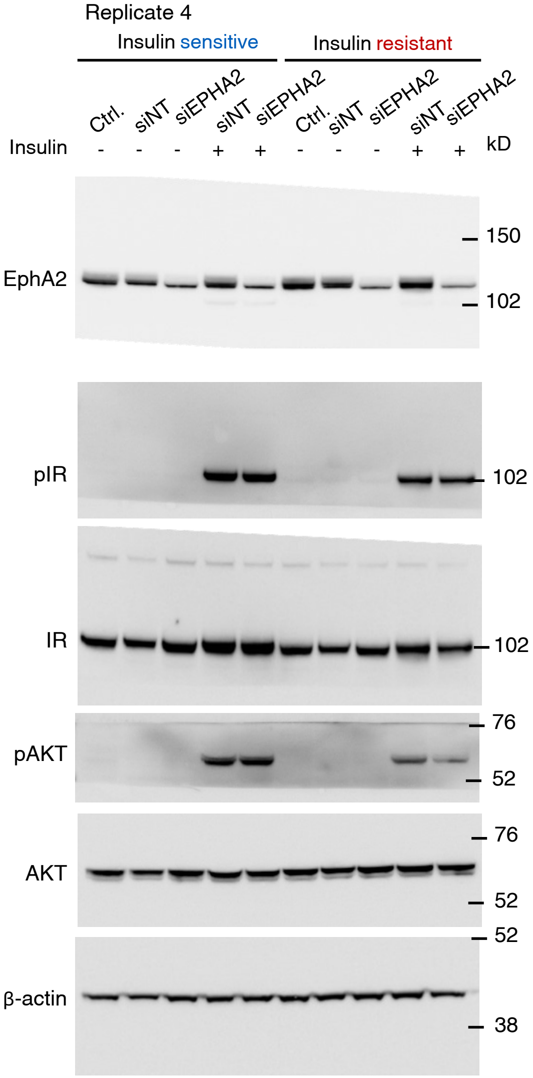

Table S1. Summary of IR interactome data upon insulin stimulation in insulin-sensitive and -resistant HepG2 IGF1R KO cells.

Table S2. Summary of phosphoproteomics data upon insulin stimulation in insulin-sensitive and -resistant cells HepG2 IGF1R KO cells.

Table S3. Summary of proteomics datasets in insulin-sensitive and -resistant HepG2 IGF1R KO cells.

Table S4. Summary of SILAC proteomics and phosphoproteomics datasets in H4IIE cells stimulated with insulin and S597.
